# An enterococcal phage-derived enzyme suppresses graft-versus-host disease

**DOI:** 10.1038/s41586-024-07667-8

**Published:** 2024-07-10

**Authors:** Kosuke Fujimoto, Tetsuya Hayashi, Mako Yamamoto, Noriaki Sato, Masaki Shimohigoshi, Daichi Miyaoka, Chieko Yokota, Miki Watanabe, Yuki Hisaki, Yukari Kamei, Yuki Yokoyama, Takato Yabuno, Asao Hirose, Mika Nakamae, Hirohisa Nakamae, Miho Uematsu, Shintaro Sato, Kiyoshi Yamaguchi, Yoichi Furukawa, Yukihiro Akeda, Masayuki Hino, Seiya Imoto, Satoshi Uematsu

**Affiliations:** 1 Department of Immunology and Genomics, Graduate School of Medicine, Osaka Metropolitan University, Osaka, Japan; 2 Division of Metagenome Medicine, Human Genome Center, Institute of Medical Science, University of Tokyo, Tokyo, Japan; 3 Department of Hematology, Graduate School of Medicine, Osaka Metropolitan University, Osaka, Japan; 4 Division of Health Medical Intelligence, Human Genome Center, Institute of Medical Science, University of Tokyo, Tokyo, Japan; 5 Department of Laboratory Medicine and Medical Informatics, Graduate School of Medicine, Osaka Metropolitan University, Osaka, Japan; 6 Department of Microbiology and Immunology, School of Pharmaceutical Sciences, Wakayama Medical University, Wakayama, Japan; 7 Division of Clinical Genome Research, Institute of Medical Science, University of Tokyo, Tokyo, Japan; 8 Department of Bacteriology I, National Institute of Infectious Diseases, Tokyo, Japan; 9 Collaborative Research Institute for Innovative Microbiology, University of Tokyo, Tokyo, Japan; 10 Reseach Institute for Drug Discovery Science, Osaka Metropolitan University, Osaka, Japan; 11 Osaka International Research Center for Infectious Diseases, Osaka Metropolitan University, Osaka, Japan

**Keywords:** Bacteriophages, Computational biology and bioinformatics, Haematological cancer

## Abstract

Changes in the gut microbiome have pivotal roles in the pathogenesis of acute graft-versus-host disease (aGVHD) after allogenic haematopoietic cell transplantation (allo-HCT)^1–6^. However, effective methods for safely resolving gut dysbiosis have not yet been established. An expansion of the pathogen *Enterococcus faecalis* in the intestine, associated with dysbiosis, has been shown to be a risk factor for aGVHD^7–10^. Here we analyse the intestinal microbiome of patients with allo-HCT, and find that *E. faecalis* escapes elimination and proliferates in the intestine by forming biofilms, rather than by acquiring drug-resistance genes. We isolated cytolysin-positive highly pathogenic *E. faecalis* from faecal samples and identified an anti-*E. faecalis* enzyme derived from *E. faecalis*-specific bacteriophages by analysing bacterial whole-genome sequencing data. The antibacterial enzyme had lytic activity against the biofilm of *E. faecalis* in vitro and in vivo. Furthermore, in aGVHD-induced gnotobiotic mice that were colonized with *E. faecalis* or with patient faecal samples characterized by the domination of *Enterococcus*, levels of intestinal cytolysin-positive *E.* *faecalis* were decreased and survival was significantly increased in the group that was treated with the *E. faecalis*-specific enzyme, compared with controls. Thus, administration of a phage-derived antibacterial enzyme that is specific to biofilm-forming pathogenic *E. faecalis*—which is difficult to eliminate with existing antibiotics—might provide an approach to protect against aGVHD.

## Abstract (web summary)

An analysis of the intestinal microbiome of people who have undergone allogenic haematopoietic cell transplantation shows that an enzyme derived from a bacteriophage has specific antibacterial activity against *Enterococcus faecalis*, and suppresses *E. faecalis*-associated graft-versus-host disease.

## Main

Acute graft-versus-host disease (aGVHD) is a life-threatening complication of allogenic haematopoietic cell transplantation (allo-HCT) for haematological diseases^11^. Patients with allo-HCT undergo many clinical interventions, such as chemotherapy, radiation and antibiotic therapy, which can affect the pathogenesis of aGVHD^12^. Several lines of evidence suggest that the microbiome has a considerable effect on aGVHD^1,2^. In the early 1970s, mice undergoing allo-HCT in germ-free conditions were found to have a lower occurrence of GVHD than conventional mice^3,4^. With advances in intestinal microbial analyses, disease-related alterations of the intestinal environment—such as dysbiosis—have been reported to be closely associated with aGVHD symptoms in clinical studies^5,6^. Under dysbiosis, some symbiotic commensal bacteria acquire pathogenic characteristics, proliferate and become directly involved in the onset and progression of the disease^13^. Such bacteria are known as ‘pathobionts’, and examples include adherent invasive *Escherichia coli* in Crohn’s disease and *Clostridium ramosum* in obesity and diabetes mellitus^14,15^. The enterococci, which are Gram-positive, facultative anaerobes, are representative pathogens detected in patients. The species *Enterococcus faecalis* and *Enterococcus faecium* are prominent causes of multidrug-resistant infections^16^, and are responsible for bloodstream infections in patients with allo-HCT^16,17^. Previous studies have also revealed the high incidence of *Enterococcus* domination in the intestine, which has been associated with aGVHD occurrence and higher mortality rates among patients with allo-HCT^7–10^. In particular, one study showed that lactose-demanding *Enterococcus* proliferated in the intestine of patients with allo-HCT, and *E. faecalis* was associated with the severity of GVHD in gnotobiotic mouse models^18^. In this regard, eliminating enterococci, especially *E. faecalis*, in the intestine of patients with allo-HCT is crucial. Faecal microbiota transplantation (FMT) can help to resolve dysbiosis. However, the positive effects of FMT on aGVHD have not been confirmed^19–22^. In addition, the safety of FMT has become a serious concern because of the occurrence of drug-resistant bacteria and the potential transmission of norovirus infection by FMT^23,24^. Therefore, performing FMT in immunocompromised patients with aGVHD can be harmful. The use of antibiotics against intestinal pathobionts also risks promoting dysbiosis by the killing of beneficial bacteria. Thus, to attenuate the increase in enterococci, developing effective and safe methods to specifically manipulate these organisms is essential for suppressing the pathogenesis of aGVHD. Bacteriophage (phage) therapy is thought to be an alternative method for eliminating pathobionts because phages infect their host bacteria specifically. We previously showed that metagenome-analysis-based information about host bacteria–phage associations helped with the detection of phage-derived antibacterial enzymes that specifically regulate pathobionts^25^. These methods have promising applications for eliminating Gram-positive bacteria.

Here we present a strategy for suppressing *E. faecalis*-associated aGVHD using a bactericidal enzyme, endolysin. We longitudinally screened the intestinal bacteriome of patients with allo-HCT and isolated *Enterococcus* species from faecal samples. Using shotgun sequencing data, we identified an antibacterial enzyme derived from *E. faecalis*-specific phages. Our results suggest that administering an *E. faecalis*-specific antibacterial enzyme suppresses the induction of aGVHD.

## Domination of *Enterococcus* in allo-HCT

Sixty-four transplant cases were at first enrolled in our study, but 18 were subsequently excluded because of early withdrawal from the study or rapid worsening of their condition. The remaining 46 transplant cases included in our analysis provided at least two sequential faecal samples. One patient was recruited into our study twice because of having undergone two transplants during our study period as a result of early leukaemia relapse after the first allo-HCT. The median age of the patients was 54.5 years (range: 19–72), and 25 (54.3%) patients were male. The patients had the following underlying diseases: 26 patients (56.5%) had acute leukaemia, 12 (26.1%) had myelodysplastic syndrome or myeloproliferative neoplasms, 7 (15.2%) had malignant lymphoma and one (2.2%) had another disease. With regard to transplants, 10 (21.7%) cases were transplanted from matched related donors, 7 (15.2%) were from matched unrelated donors, 11 (23.9%) were from cord blood, 17 (37.0%) were from haploidentical donors and one (2.2%) was from a human leukocyte antigen (HLA)-mismatched unrelated donor. Graft types included bone marrow in 8 (17.4%) cases, peripheral blood stem cells in 27 (58.7%) cases and cord blood in 11 (23.9%) cases. Myeloablative, reduced intensity and nonmyeloablative conditioning therapies were performed in 24 (52.2%), 21 (45.7%) and one (2.2) cases, respectively. Fourteen (30.4%) cases received total body irradiation as part of their conditioning therapeutic regimen. In total, fluoroquinolone, sulfamethoxazole–trimethoprim, isoniazid and macrolide were prophylactically administered in 44 (95.7%), 39 (84.5%), 3 (6.5%) and 2 (4.3%) cases, respectively, before the conditioning therapies. As empiric or target therapies, cefems (cefepim or cefozopran), piperacillin–tazobactam, carbapenems (meropenem or doripenem), glycopeptides (vancomycin or teicoplanin) or lipopeptides (daptomycin) and metronidazole were administered in 21 (45.7%), 26 (56.5%), 17 (37.0%), 28 (60.9%) and 3 (6.5%) cases, respectively, during the sample collection periods (Supplementary Table 1). The detailed characteristics of each case are summarized in Supplementary Table 2.

**Extended Data Fig. 1:**
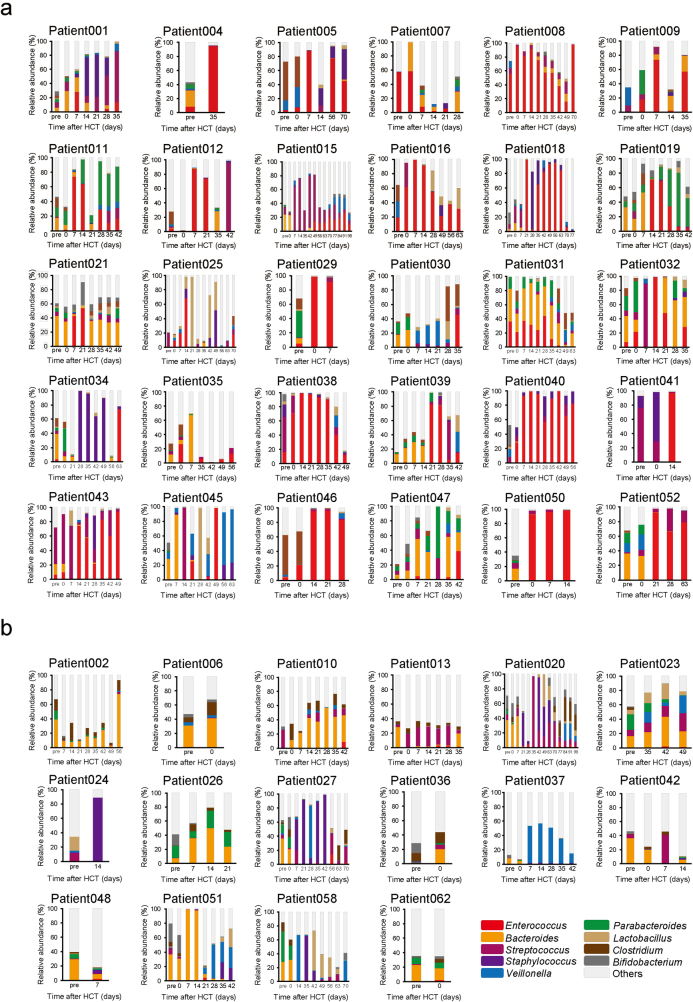
Gut bacterial composition in patients with allo-HCT. **a**,**b**, Gut bacterial composition at the genus level based on the relative abundance of operational taxonomic units for faecal samples from patients with allo-HCT. **a**, Cases with *Enterococcus* domination (defined as >25% of the genus *Enterococcus*) at any time point. **b**, Cases without *Enterococcus* domination. Source Data

A total of 317 faecal samples were obtained from the 46 cases. We extracted DNA from all of these samples and analysed their intestinal microbial composition by 16S ribosomal RNA (rRNA) gene sequencing (Extended Data Fig. 1a,b). *Enterococcus* domination, which was defined as a relative abundance of the *Enterococcus* genus of more than 25% in a faecal sample, on the basis of previous studies^7,18^, was observed in 89 samples from 30 (65.2%) cases. Analysis of clinical predictors by univariate Cox proportional hazards regression revealed that acute leukaemia was significantly associated with a higher risk of *Enterococcus* domination than other diseases (hazard ratio, 2.48; 95% confidence interval, 1.13 to 5.45, *P* = 0.024) (Supplementary Table 3). No other clinical variables were significantly associated with the cumulative incidence of *Enterococcus* domination. Thus, consistent with previous research^7,18^, *Enterococcus* domination was characterized in our patients with allo-HCT.

## Characterization of *E. faecalis*

**Fig. 1:**
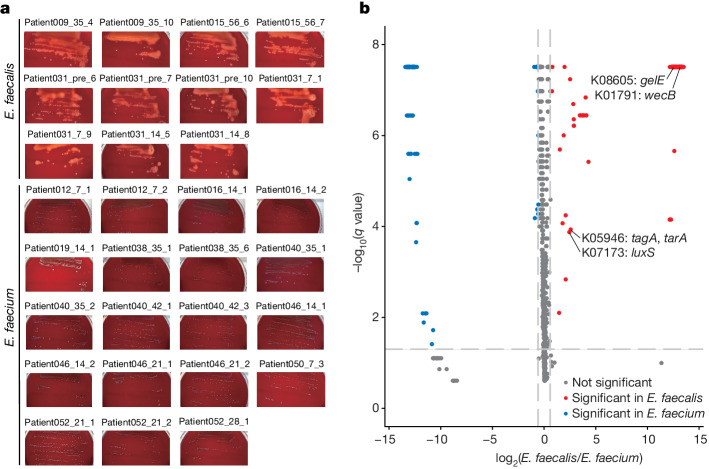
Characterization of enterococcal strains in patients with allo-HCT. **a**, Detection of functional haemolytic activity. Representative image from two independent experiments. **b**, Volcano plot comparing the KO terms of *E. faecalis* and *E. faecium* strains. Circles coloured blue and red indicate significantly different pathways (more than twofold, *q* < 0.05, according to the Wilcoxon rank-sum test (two-sided)) between *E. faecalis* and *E. faecium* strains. Source Data

**Extended Data Fig. 2:**
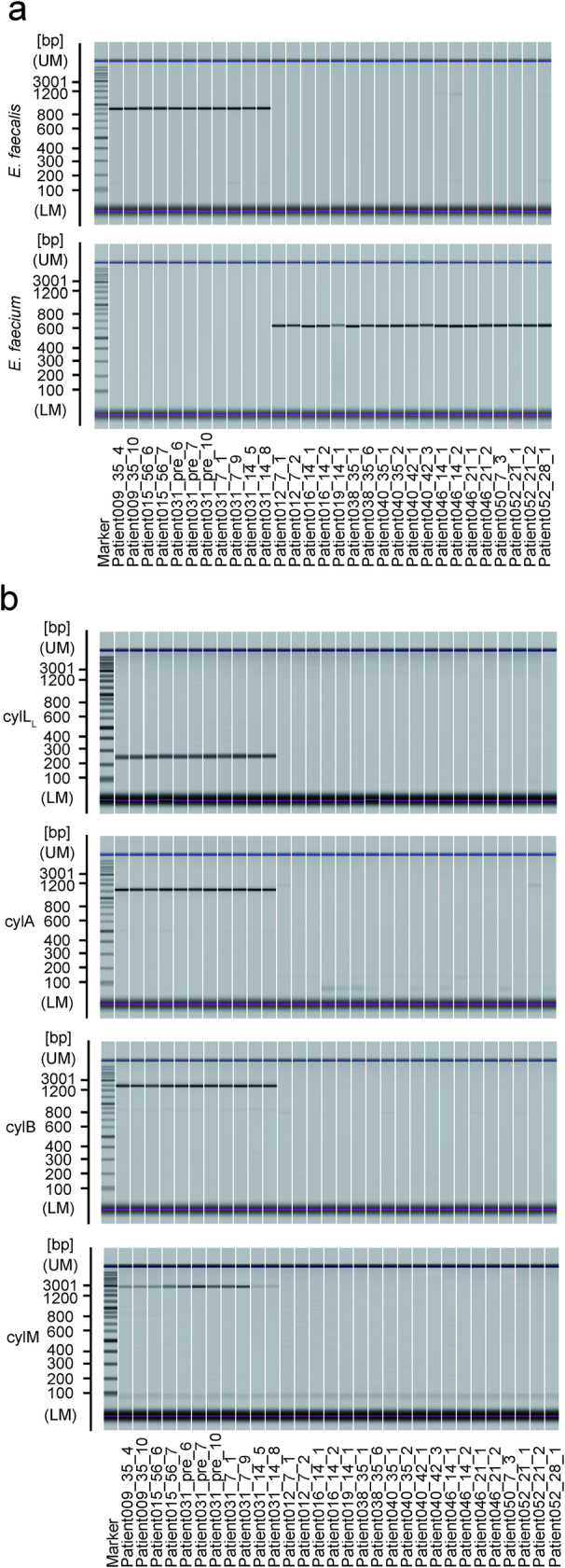
Cytolysin-associated genes in *E. faecalis* and *E. faecium* strains. **a**, Detection of *E. faecalis* and *E. faecium* strains isolated from faecal samples of allo-HCT patients by PCR. A representative image obtained from two independent experiments is shown. **b**, Detection of *cylL*_*L*_, *cylA*, *cylB*, and *cylM* in each strain by PCR. A representative image obtained from two independent experiments is shown.

We next isolated 30 enterococci from faecal samples characterized by *Enterococcus* domination, and performed metagenomic analysis. We identified 11 strains of *E. faecalis* and 19 strains of *E. faecium* (Extended Data Fig. 2a). Several studies have shown that the colonization of multidrug-resistant bacteria, such as vancomycin-resistant enterococci (VRE), correlated with increased mortality among patients with allo-HCT^17^. We therefore performed antimicrobial susceptibility testing for the 30 isolated enterococci. Notably, all *E. faecalis* and *E. faecium* strains were sensitive to vancomycin, teicoplanin, daptomycin and linezolid, indicating that these bacterial strains were not VRE, and were consistent with the standard epidemiological features of enterococcal strains isolated in Japan^26^ (Extended Data Table 1). Despite the multidrug resistance of enterococci, cytolysin, which is an exocrine protein that is associated with haemolysin, contributes to the severity of enterococcal disease in humans^27–29^ and a number of animal models^30–33^. We detected cytolysin-associated genes, such as *cylL*_*L*_, *cylA*, *cylB* and *cylM*, in all *E. faecalis* strains, but not in *E. faecium* strains (Extended Data Fig. 2b). In addition, all of the isolated cytolysin-positive *E. faecalis* strains had functional haemolytic activity (Fig. 1a and Supplementary Fig. 1), indicating that highly virulent *E. faecalis* was dominant in patients with allo-HCT. Furthermore, although Kyoto Encyclopaedia of Genes and Genomes (KEGG) orthology (KO) terms related to biofilm formation were identified for both species, some terms, such as K01791, K07173, K05946 and K08605 were more frequently detected in *E. faecalis* (Fig. 1b). Because it is estimated that biofilm-forming bacteria are 10 to 1,000 times more resistant to antibiotics than are planktonic bacteria^34,35^, we hypothesized that cytolysin-positive virulent *E. faecalis* survive and proliferate by forming biofilms in the intestine of patients with allo-HCT, and thereby resist the effects of the various antibiotics administered during the treatment process.

## A phage-derived anti-bactericidal enzyme

**Fig. 2:**
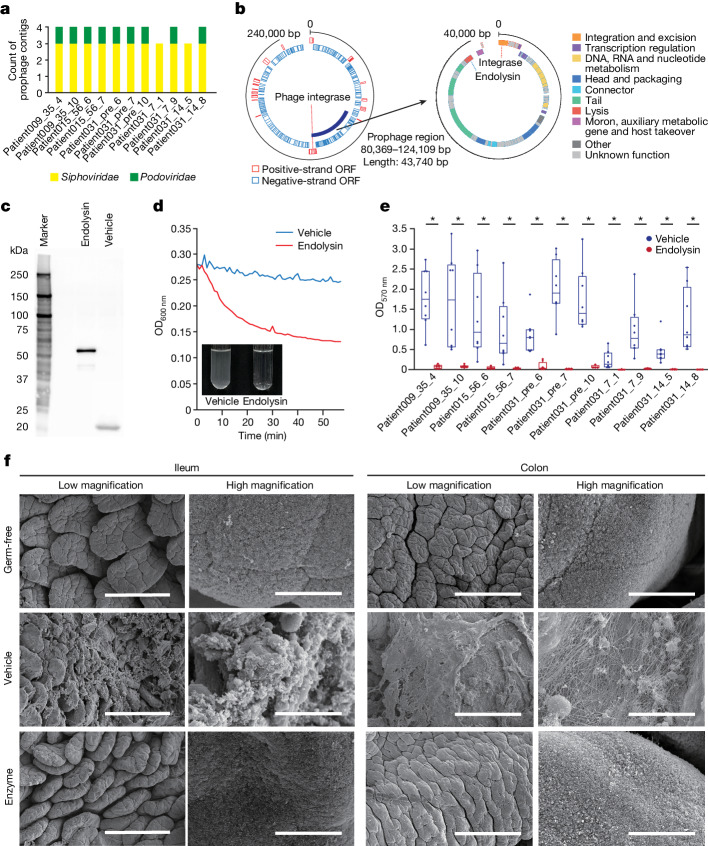
Lysis of biofilms by a phage-derived antibacterial enzyme derived from an *E. faecalis*-specific prophage. **a**, Number of detected prophage sequences and their viral taxa in each *E. faecalis* strain. **b**, Representative bacterial contig showing the whole sequence of a viral contig classified as a putative member of the *Podoviridae* containing endolysin in an *E. faecalis* strain obtained from Patient031_14_8. **c**, Expression and purification of endolysin by western blotting. Representative blot obtained from two independent experiments. **d**, Bacteriolytic capacity of endolysins against an *E. faecalis* strain obtained from Patient031_14_8. Representative experiment and image from two independent experiments. OD_600 nm_, optical density at 600 nm. **e**, Mature biofilm assay with crystal violet staining 24 h after incubation with endolysin or vehicle, including His-SUMO (*n* = 8 in each group). *P = 0.0001554. Significance was determined using the Wilcoxon rank-sum test (two-sided). The line inside the box represents the median. The whiskers represent the range of points up to 1.5 times the interquartile range. Data are representative of two independent experiments. OD_570 nm_, optical density at 570 nm **f**, Oral administration of endolysin or vehicle in germ-free mice (*n* = 2) or gnotobiotic mice that were mono-colonized with *E. faecalis* (*n* = 2). Representative scanning electron microscope images of the small and large intestines. Scale bars, 200 µm (low-magnification images); 5 µm (high-magnification images). Source Data

**Extended Data Fig. 3:**
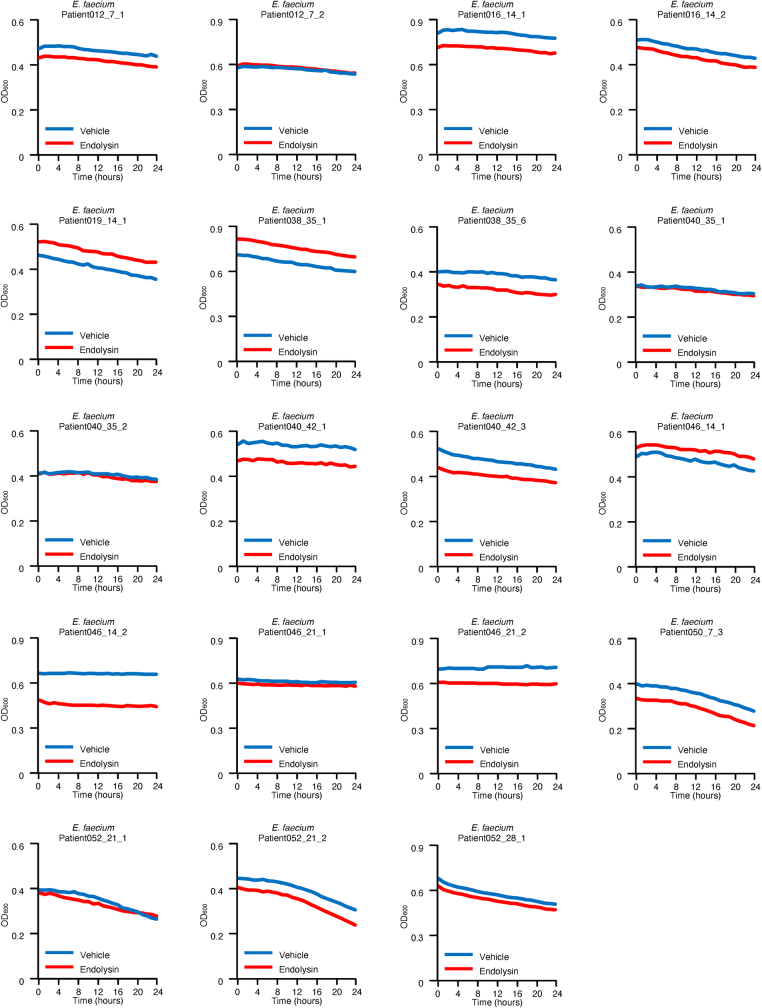
Analysis of the bacteriolytic capacity of endolysins against *E. faecium.* Bacteriolytic capacity of endolysins against *E. faecium* isolated from the faeces of patients with allo-HCT. A representative experiment from two independent experiments is shown. Source Data

**Extended Data Fig. 4:**
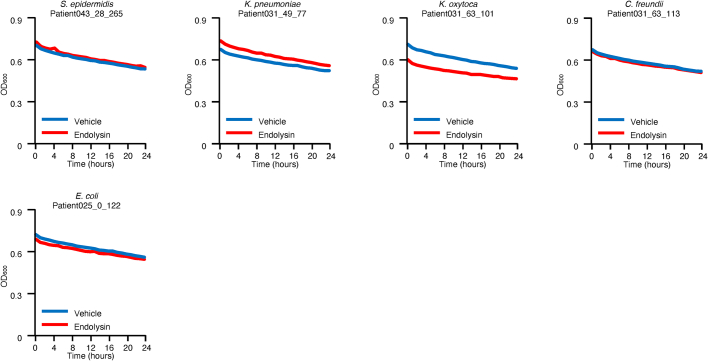
Analysis of the bacteriolytic capacity of endolysins against intestinal bacteria. Bacteriolytic capacity of endolysins against *S. epidermidis*, *K. pneumoniae*, *K. oxytoca*, *C. freundii* and *E. coli* isolated from the faeces of patients with allo-HCT. A representative experiment from two independent experiments is shown. Source Data

**Extended Data Fig. 5:**
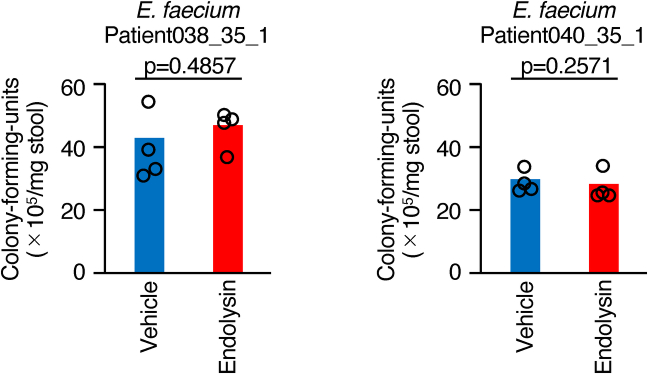
Analysis of *E. faecium* CFU in colonized gnotobiotic mice. CFU of *E. faecium* strain Patient038_35_1 and *E. faecium* strain Patient040_35_1 in faecal samples. Each dot represents the data from one faecal sample. *n* = 4 for endolysin-treated mice mono-colonized with *E. faecium* strain Patient038_35_1; *n* = 4 for vehicle-treated mice mono-colonized with *E. faecium* strain Patient038_35_1; *n* = 4 for endolysin-treated mice mono-colonized with *E. faecium* strain Patient040_35_1; and *n* = 4 for vehicle-treated mice mono-colonized with *E. faecium* strain Patient040_35_1. Significance was determined using the Wilcoxon rank-sum test (two-sided). Source Data

Because *E. faecalis*, which expands in the intestine of patients with allo-HCT, is considered to be one of the pathobionts associated with the severity of aGVHD, specific elimination of this organism is desirable to suppress aGVHD^8,9,18^. However, biofilms exhibit strong resistance to antibiotics through mechanisms such as reduced antibiotic permeability, sequestration of antibiotics and the presence of persister cells^36–38^. Hence, it is extremely difficult to eliminate *E. faecalis* with existing antibiotics. Endolysins are hydrolytic enzymes produced by phages that dissolve the bacterial cell wall to release progeny phages at the end of the replication cycle^39^. These enzymes hydrolyse the peptidoglycan layer and kill the host bacteria^40^. Compared with conventional broad-spectrum antibiotics, endolysins confer advantages such as high host specificity, rapid lysis of target bacteria, a low risk of resistance, synergy with different antimicrobial agents and the ability to function effectively on biofilms and mucosal surfaces^41–46^. In a previous study, we successfully identified novel endolysins in *Clostridioides difficile* prophage sequences from metagenomic data, and confirmed their bactericidal activity against *C. difficile* both in vitro and in vivo^25^. Therefore, we next analysed shotgun sequence data relating to *E. faecalis* strains (Fig. 1a and Extended Data Fig. 2a,b), and extracted prophage sequences. Up to four prophage sequences classified as *Siphoviridae* or *Podoviridae* were identified from each set of sequence data (Fig. 2a). Open reading frames (ORFs) homologous to known endolysins in prophage genomes were screened (Fig. 2b). We identified nine endolysins from the metagenomic data for the *E. faecalis* strains shown in Fig. 1a–c, all of which were annotated as WP_002399372 (*E* value = 0) (Supplementary Fig. 2). In general, endolysins consist of two domains—an enzymatically active domain and a cell-wall-binding domain—that recognize specific cell-surface features. The enzymatically active domain of the detected endolysins was annotated as GH25 (glycoside hydrolase family 25)_LysA-like, which is an endo-*N*-acetylmuramidase (muramidase) that degrades bacterial cell walls by catalysing the hydrolysis of 1,4-β-linkages between *N*-acetylmuramic acid and *N*-acetyl-d-glucosamine residues. The cell-wall-binding domain of the detected endolysins was annotated as ZoocinA_TRD (target recognition domain). We generated a His-SUMO-tagged endolysin (Fig. 2c and Supplementary Fig. 3), and found that it had effective lytic activities in vitro (Fig. 2d and Supplementary Fig. 4). In addition, the biofilms from all isolated *E. faecalis* strains in this study were successfully lysed by the endolysin (Fig. 2e). Notably, the endolysin did not have effective lytic activity against *E. faecium* or other intestinal bacteria such as *Staphylococcus epidermidis*, *Klebsiella pneumoniae*, *Klebsiella oxytoca*, *Citrobacter freundii* and *E. coli* isolated from the faeces of patients with allo-HCT (Extended Data Figs. 3–5), which suggests that the endolysin is a narrow-spectrum enzyme. Furthermore, oral administration of the endolysin in gnotobiotic C57BL/6 mice that were mono-colonized with *E. faecalis* resulted in the effective lysis of *E. faecalis*-derived biofilms in the intestine (Fig. 2f and Supplementary Fig. 5). These data suggest that the endolysin detected in the *E. faecalis* prophage sequence from the metagenome data has the potential to lyse the biofilms formed by *E. faecalis* in the intestine.

## Suppression of aGVHD

**Fig. 3:**
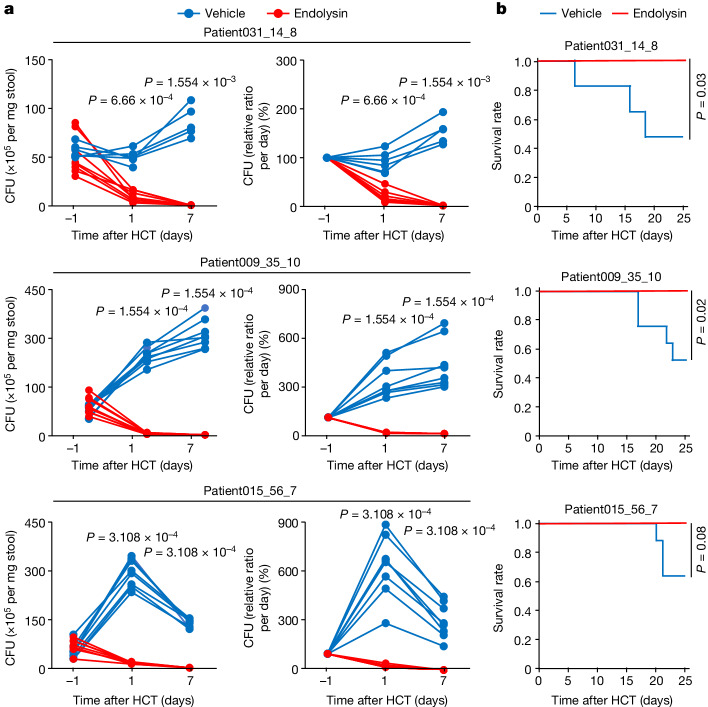
Analysis of *E. faecalis* phage-derived endolysin in *E. faecalis*-mono-colonized gnotobiotic GVHD mice. **a**, Colony-forming units (CFU) (left) and percentage CFU (right) of *E. faecalis* in faecal samples before and after transplantation. Each dot and line represent the data from one faecal sample. Significance was determined using the Wilcoxon rank-sum test (two-sided). Patient031_14_8: *n* = 8 for endolysin-treated mice, *n* = 6 for vehicle-treated mice. Patient009_35_10: *n* = 8 for endolysin-treated mice, *n* = 8 for vehicle-treated mice. Patient015_56_7: *n* = 8 for endolysin-treated mice, *n* = 7 for vehicle-treated mice. **b**, Kaplan–Meier survival plots of endolysin-treated mice and vehicle-treated mice. Significance was determined using the log-rank test (two-sided). The numbers of endolysin-treated mice and vehicle-treated mice for each of the patient samples are as in **a**. Source Data

**Fig. 4:**
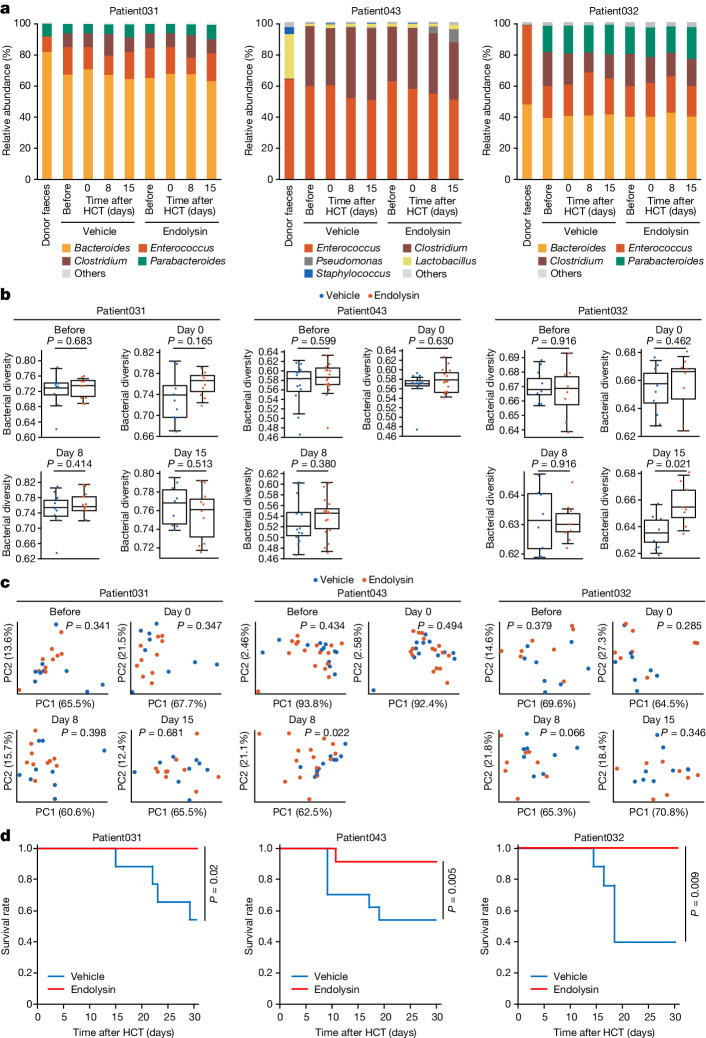
Analysis of *E. faecalis* phage-derived endolysin in humanized gnotobiotic GVHD mice. **a**, Microbial composition of the gut, on the basis of the relative abundance of operational taxonomic units at the genus level, for the donor faeces and the faecal samples from humanized mice before and after transplantation. Patient031: *n* = 10 for endolysin-treated mice, *n* = 9 for vehicle-treated mice. Patient043: *n* = 16 for endolysin-treated mice, *n* = 13 for vehicle-treated mice. Patient032: *n* = 8 for endolysin-treated mice, *n* = 8 for vehicle-treated mice. The mean of the relative abundance in each group is shown. **b**, Bacterial alpha diversities of faecal microbial communities using Pielou’s evenness index. Significance was determined using the Kruskal–Wallis test (two-sided). The line inside the box represents the median. The whiskers represent the range of points up to 1.5 times the interquartile range. There were some deaths in the day-15 Patient043 group and this group was therefore excluded. **c**, Principal coordinate analysis of the weighted UniFrac distance matrices for the faecal microbial communities. Significance was determined using a pairwise PERMANOVA. NS, not significant. Red, endolysin-treated mice; blue, vehicle-treated mice. There were some deaths in the day-15 Patient043 group and this group was therefore excluded. **d**, Kaplan–Meier survival plots of endolysin-treated mice and vehicle-treated mice. Significance was determined using the log-rank test (two-sided). Patient031: *n* = 10 for endolysin-treated mice, *n* = 9 for vehicle-treated mice. Patient043: *n* = 16 for endolysin-treated mice, *n* = 13 for vehicle-treated mice. Patient032: *n* = 8 for endolysin-treated mice, *n* = 8 for vehicle-treated mice. Source Data

**Extended Data Fig. 6:**
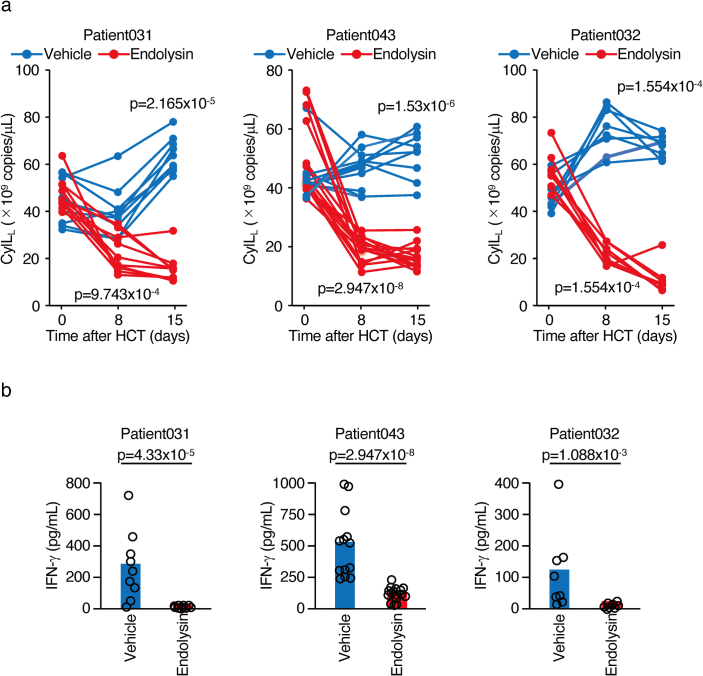
Analysis of the *E. faecalis cylL*_*L*_ gene copy number in faecal samples before and after transplantation, and IFNγ levels in the serum of *E. faecalis-*colonized gnotobiotic GVHD mice. **a**, Each dot and line represents the data from one faecal sample. Significance was determined using the Wilcoxon rank-sum test (two-sided). Patient031: *n* = 10 for endolysin-treated mice, *n* = 9 for vehicle-treated mice. Patient043: *n* = 16 for endolysin-treated mice, *n* = 13 for vehicle-treated mice. Patient032: *n* = 8 for endolysin-treated mice, *n* = 8 for vehicle-treated mice. **b**, IFNγ production in serum. Each dot represents the data from one serum sample. Significance was determined using the Wilcoxon rank-sum test (two-sided). The numbers of endolysin-treated mice and vehicle-treated mice for each of the patient samples are as in **a**. Source Data

To investigate the potential effectiveness of administering the purified endolysin against *E. faecalis* as an aGVHD suppression strategy, we used gnotobiotic C57BL/6 mice mono-colonized with *E. faecalis* (Supplementary Fig. 6a). After administration of busulfan and cyclophosphamide, bone marrow cells and splenic T cells were intravenously injected into the gnotobiotic mice to induce aGVHD (Supplementary Fig. 6a). The levels of *E. faecalis* in the faeces of the endolysin-treated gnotobiotic mice were significantly lower than those in the vehicle-treated gnotobiotic mice (Fig. 3a). Furthermore, the endolysin was effective against aGVHD (Fig. 3b). These findings indicated that the *E. faecalis*-derived endolysin regulated aGVHD by reducing the number of intestinal *E. faecalis*. We also analysed GVHD-induced gnotobiotic C57BL/6 mice colonized with a human faecal sample that was characterized by the domination of *Enterococcus* (Fig. 4a and Supplementary Fig. 6b). Levels of the cytolysin-associated gene *cylL*_*L*_ in the faeces of the endolysin-treated humanized mice were consistently and significantly lower than those of the vehicle-treated humanized mice (Extended Data Fig. 6a). Before (pre-transplantation and day 0) and after (day 8 and day 15) transplantation, the microbial communities showed no consistent significant differences between the two groups (Fig. 4b,c). However, the endolysin-treated humanized mice showed significantly decreased levels of interferon gamma (IFNγ) in the serum (Extended Data Fig. 6b) and an increased survival rate compared with the vehicle-treated humanized mice (Fig. 4d). Together, these data suggest that the endolysin detected in the *E. faecalis* prophage sequences from the metagenome data has potential clinical applications for aGVHD.

## Discussion

In this study, we analysed the intestinal bacteriome of patients with allo-HCT and revealed the domination of *Enterococcus*. An endolysin identified from *E. faecalis* prophage sequencing data was shown to lyse biofilm-forming *E. faecalis*. Administration of this endolysin eradicated cytolysin-positive virulent *E. faecalis* in the intestine and suppressed the exacerbation of aGVHD.

In our study, *Enterococcus* domination was observed in 65.2% of patients with allo-HCT (Extended Data Fig. 1a,b), and acute leukaemia was significantly associated with a high incidence of *Enterococcus* domination (Supplementary Table 3), consistent with a previous study^7^. Because patients with acute leukaemia are administered more cycles of highly intensified chemotherapy and broad-spectrum antibiotics before allo-HCT than are patients with other diseases, in accordance with previous studies^47^, gut dysbiosis and dysfunction of the intestinal barrier might have been induced. A previous report showed that bacteria living in biofilms are considerably more resistant to antibiotics than are planktonic bacteria^48^; therefore, biofilm-forming enterococci might have been able to remain dominant in the intestinal tracts of patients with allo-HCT.

Antimicrobial susceptibility testing confirmed that all isolated *E. faecalis* and *E. faecium* strains in this study were not VRE (Extended Data Table 1). In addition, VRE strains were not isolated through the culturing of 223 faecal samples dominated by *Enterococcus* on VRE-selective agar. Thus, it is unlikely that acquiring resistance to glycopeptide or oxazolidine antibiotics—which are the antibiotics most frequently administered during allo-HCT to prevent infection—contributes to the development of *Enterococcus* domination in the intestinal tract. However, all isolated *E. faecalis* strains, but not *E. faecium* strains, possessed cytolysin genes (Extended Data Fig. 2b), indicating that these bacteria were highly virulent. In fact, a high incidence of *Enterococcus* domination in the intestine has been shown to correlate with aGVHD occurrence and the higher mortality rates among patients with allo-HCT^18^. Therefore, inhibiting biofilm formation and eliminating cytolytic *E. faecalis* would be an effective clinical approach in patients with allo-HCT.

Characterization of the isolated *E. faecalis* strains from patients with allo-HCT revealed an increase in the levels of several KO terms associated with biofilm formation, including K01791, K05496, K07173 and K08605, compared with the *E. faecium* strains (Fig. 1b). The ability of *E. faecalis* to readily colonize the naive gut epithelium by forming biofilms^49^ has been previously shown in a germ-free mouse model. K01791 and K05496 are KO terms associated with the *wecB* and *tagA* genes, respectively, which play a dedicated role in polysaccharide biosynthesis^50^. K07173 is a KO term associated with the *luxS* genes, which are involved in quorum sensing and biofilm formation^51,52^. K08605 is a KO term associated with the *gelE* genes, which are essential for producing gelatinase and contribute to biofilm formation^53,54^. A previous study showed that the metalloprotease GelE produced by *E. faecalis* impaired the function of the intestinal epithelial barrier and induced chronic inflammation in IL-10^−/^^−^ and TNF^ΔARE/Wt^ mice^55^, which suggests that, in addition to cytolysin, GelE might be a clinical target for aGVHD.

**Extended Data Fig. 7:**
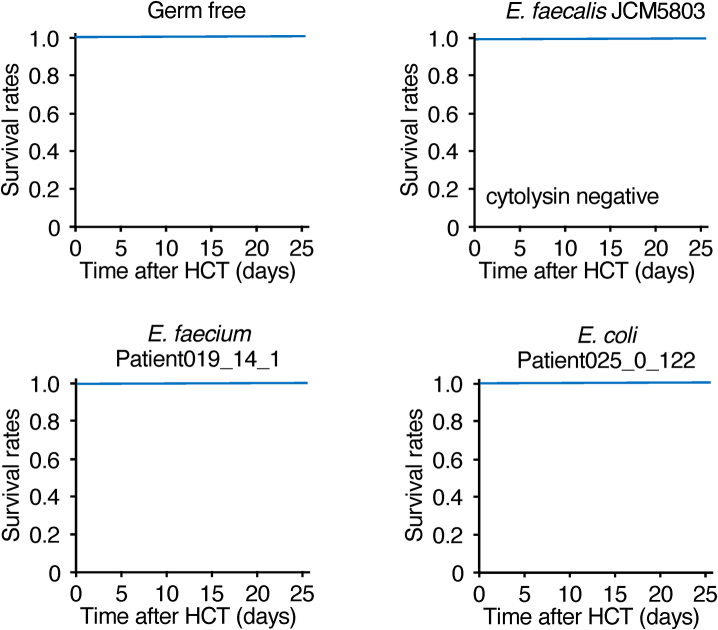
Analysis of survival rates in germ-free mice and in cytolysin-negative *E. faecalis*-, *E. faecium*- and *E. coli*-mono-colonized gnotobiotic GVHD mice. Kaplan–Meier survival plots of germ-free mice (*n* = 7) and mice mono-colonized with *E. faecalis* strain JCM5803 (*n* = 8), *E. faecium* strain Patient019_14_1 (*n* = 8), and *E. coli* strain Patient025_0_122 (*n* = 7). Source Data

Because *E. faecalis* is a Gram-positive bacterium, it was predicted that endolysins, which specifically hydrolyse peptidoglycan and are involved in the formation of biofilms, would decrease the domination of *E. faecalis* in the intestine. Several studies have reported the identification of endolysins from meta-analyses of phage sequences^25,56^. In our previous study, we showed that metagenome-analysis-based information about host bacteria–phage associations is useful for detecting phage-derived antibacterial enzymes that specifically control pathobionts^25^. In our current study, we detect a unique endolysin from *E. faecalis* prophage sequencing data (Fig. 2b and Supplementary Fig. 2), and provide evidence of its lytic activity against *E. faecalis* strains—but not *E. faecium* strains—isolated from patients with allo-HCT (Fig. 2d and Extended Data Fig. 3). Notably, the identified endolysin eradicated the formation of biofilms (Fig. 2e,f) and protected against aGVHD (Figs. 3b and 4d). Furthermore, aGVHD-related mortality rates were not increased in cases associated with the cytolysin-negative *E. faecalis* strain JCM5803, *E. faecium* strain Patient019_4_1, and *E. coli* strain Patient025_0_122 (Extended Data Fig. 7). Future research will be required to determine whether this therapeutic approach is effective for patients with aGVHD.

When considering the possible clinical applications of the endolysin against *E. faecalis*, specificity of the enzyme for *E. faecalis* would be a desirable characteristic. Indeed, the lytic spectrum of an endolysin ranges from species-specific to targeting multiple genera. A previous study showed that enterococcal phage lysins (for example, PlyV12 and EFAP-1) have a broad-spectrum killing ability^57^. However, in our study, the endolysin lysed *E. faecalis*, but not *E. faecium* or other intestinal bacteria (Fig. 2d–f and Extended Data Figs. 3–5). Therefore, as well as the *E. faecalis*-specific endolysin LysEF-P10 previously reported^58^, our identified endolysin might be a narrow-spectrum enzyme, which would make it an ideal candidate to eliminate *E. faecalis*.

In the present study, of the 30 *Enterococcus*-dominant cases, 7 were aGVHD-positive and cytolysin-positive, 4 were aGVHD-positive and cytolysin-negative, 3 were aGVHD-negative and cytolysin-positive and 16 were aGVHD-negative and cytolysin-negative (*P* < 0.0147 by two-tailed Fisher’s exact test) (Supplementary Table 2). Although the sample size is small, our study is the first—to our knowledge—to show not only an experimental but also a clinical association between faecal cytolysin positivity and the development of aGVHD. Thus, the relationship between the cytolysin positivity of *E. faecalis* and the development of aGVHD in patients with allo-HCT should be a key consideration in the design of therapeutic strategies.

**Extended Data Fig. 8:**
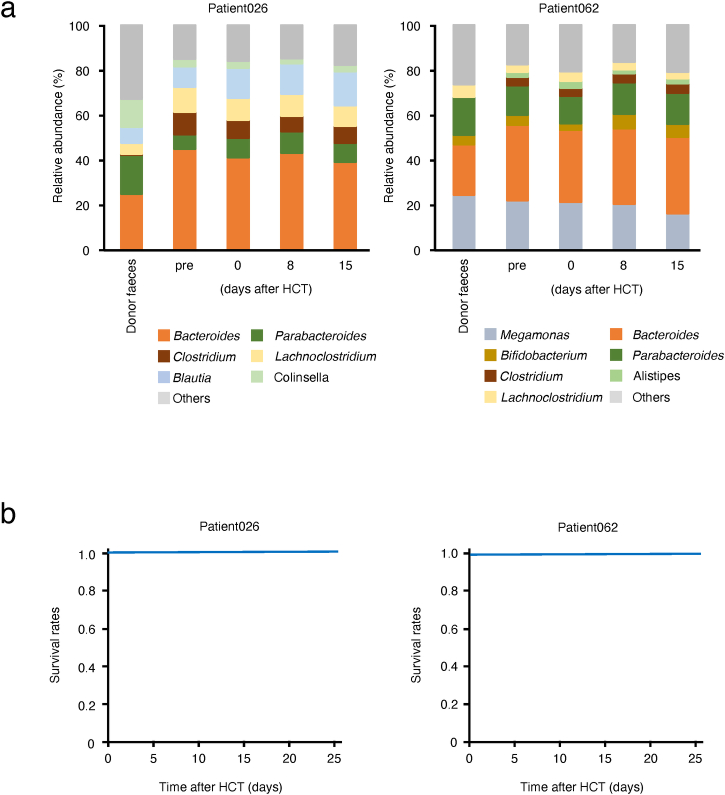
Analysis of the gut microbial composition and survival rates of humanized GVHD mice. **a**, Gut microbial composition based on the relative abundance of operational taxonomic units at the genus level for the donor faeces and the faecal samples from humanized mice before and after transplantation. *n* = 8 samples for Patient026, and *n* = 8 samples for Patient062. **b**, Kaplan–Meier survival plots of mice colonized with faeces from Patient026 (*n* = 8) and mice colonized with faeces from Patient062 (*n* = 8). Source Data

This study has several limitations. To investigate the effect of endolysin on aGVHD, but not chronic GVHD, we administered endolysin for only three weeks and observed survival rates for one month. Future studies could assess whether cytolysin-positive *E. faecalis* is effective against chronic GVHD. In addition, the small sample size of our current cohort study precludes a detailed investigation of cytolysin-positive *E. faecalis* and the severity of aGVHD in patients with allo-HCT. However, according to previous reports, *E. faecalis*-derived cytolysin lyses bacterial cells, as well as eukaryotic cells, in response to quorum-sensing signals^59^, indicating that cytolysin-positive *E. faecalis*, not cytolysin-negative *E. faecalis*, has a greater ability to proliferate as a result of the suppression of competing bacteria in the intestinal tract of patients with allo-HCT. In the present study, aGVHD-related mortality was increased in cases with cytolysin-positive *E. faecalis* and an *Enterococcus*-dominated gut microbiome, but not in cases with cytolysin-negative *E. faecalis* or a gut microbiome that is not dominated by *Enterococcus* (Fig. 3b and Extended Data Figs. 7 and 8). Future studies will be required to address whether cytolysin-positive *E. faecalis* is involved in the pathogenesis of aGVHD in patients with allo-HCT. Furthermore, although we did not detect VRE in all enterococci in this study, the presence of VRE has been shown to be inevitable in patients with allo-HCT^16,17^. Future studies are required to address whether *E. faecalis*-derived endolysin is effective against VRE.

In conclusion, we report a new strategy for regulating aGVHD that uses an endolysin derived from *E. faecalis*. As well as aGVHD, cytolytic *E. faecalis* in the intestine has been shown to be associated with the severity of liver disease and with mortality in patients with alcoholic hepatitis^60^. *E. faecalis* also causes other antibiotic-refractory infections, such as infective endocarditis, endophthalmitis and catheter-related urinary tract infections, by forming biofilms. Thus, our findings raise the possibility of developing new treatments for cytolytic *E. faecalis*-associated diseases, represented by aGVHD.

## Methods

### Patient cohort

An observational cohort consisting of allo-HCT recipients was recruited from patients in the Department of Haematology at Osaka Metropolitan University Hospital. Patients with haematological diseases who were undergoing allo-HCT from January 2019 to June 2020 were included. For each patient, sequential faecal samples were collected before stem cell transplantation (within 14 days before the initial date of conditioning therapy), and then weekly (at day 0 ± 3, day 7 ± 3, day 14 ± 3 and so on) until day 98 ± 3, or hospital discharge. The date of stem cell transfusion was defined as day 0. Sample collection was skipped when patients could not provide stool specimens because their general condition had worsened. This protocol was approved by the Ethics Committee of Osaka Metropolitan University and the Institute of Medical Science, The University of Tokyo, and signed informed consent was obtained from each participant (4188 and 30-92-B0320).

### Antibiotics in patients with allo-HCT

Prophylactic fluoroquinolone and sulfamethoxazole–trimethoprim were started before conditioning therapy, except in patients with drug allergies or who had already received systemic antibiotics. Isoniazid or macrolide were given in addition to patients with latent tuberculosis infection or chronic sinusitis, respectively. Sulfamethoxazole–trimethoprim was stopped two days before stem cell transplantation, in consideration of its suppressive effect on the bone marrow. Our antibiotic administration strategy was based on the guidelines of the Infectious Diseases Society of America^61^, which specified that in the case of febrile neutropenia (defined as an axillary temperature higher than 37.5 °C and a neutrophil count lower than 0.5 × 10^9^ per litre), fluoroquinolone was switched to a cefem or to piperacillin–tazobactam as the first-line therapy. When fever persisted, the antibiotics were further changed to other agents, such as carbapenems, as the second-line treatment. Glycopeptides or lipopeptides were also administered when a catheter-related infection, a skin or soft-tissue infection, pneumonia or haemodynamic instability was suspected. In addition, targeted antibiotics were administered according to the culture-based results or clinical signs, such as metronidazole for *C. difficile* enteritis.

### Extraction of bacterial DNA

After collection, the patient faecal samples were immediately stored at 4 °C under anaerobic conditions, shipped to the laboratory, then divided and stored in RNA later (Invitrogen), 40% glycerol and anaerobic culture medium (2% Lab-Lemco powder (Kanto Chemical Co.), 0.1% l-cysteine (Nacalai Tesque), 0.045% KH_2_PO_4_ (Nacalai Tesque), 0.09% NaCl (Nacalai Tesque), 0.045% [NH_4_]_2_SO_4_ (Nacalai Tesque), 0.0045% CaCl_2_ (Nacalai Tesque), 0.0045% MgSO_4_ (Nacalai Tesque) and 40% glycerol (Nacalai Tesque) in 1 l distilled water) at a 16-fold dilution (w/v) at −80 °C until use. Bacterial DNA extraction from the faecal samples was performed as previously described^25^. In brief, faecal samples stored in RNA later were homogenized in 1 ml SM-plus buffer (100 mM NaCl, 50 mM Tris-HCl (pH 7.4) (Nacalai Tesque), 8 mM MgSO_4_·7H_2_O (Nacalai Tesque), 5 mM CaCl·2H_2_O (Nacalai Tesque) and 0.01% (w/v) gelatine in distilled water) by vortex mixing, and were then passed through a 100-µm cell strainer. SM-plus buffer was passed through a 0.22-µm syringe filter before use at each step. To extract DNA, the samples were incubated with 1 ml SM-plus buffer containing 20 mM EDTA (Nacalai Tesque), 100 µg ml^−1^ recombinant human lysozyme (Sigma-Aldrich) and 0.5 U ml^−1^ achromopeptidase (Wako Pure Chemicals) at 37 °C for 1 h. Then, supernatant samples were further incubated with a 1/400 volume of 20 mg ml^−1^ proteinase K (Nacalai Tesque) and a 1/20 volume of 10% sodium dodecyl sulfate (Nacalai Tesque) at 55 °C for 1 h. Next, the samples were added to an equal volume of phenol–chloroform–isoamyl alcohol (Nacalai Tesque) and mixed vigorously. After centrifugation at 16,000*g* for 5 min, the aqueous phase of the samples was transferred to new tubes, followed by chloroform (Nacalai Tesque) extraction. Again, the aqueous phase of the samples was transferred to new tubes and mixed with a 1/10 volume of 3 M sodium acetate (Nacalai Tesque) and an equal volume of isopropanol (Nacalai Tesque). The samples were centrifuged at 16,000*g* for 15 min. After discarding the supernatants, the pellets were washed with 70% ethanol (Nacalai Tesque) and centrifuged at 16,000*g* for 5 min. The supernatants were removed completely, and the pellets were air-dried for 5 min. The DNA was resuspended in 10 mM Tris-HCl buffer (pH 8.0) (Nacalai Tesque).

### 16S rRNA gene analysis

The 16S rRNA V3–V4 region was PCR-amplified using specific primers (forward primer: 5′-ACACGACGCTCTTCCGATCTCCTACGGGNGGCWGCAG-3′, reverse primer: 5′-GACGTGTGCTCTTCCGATCTGACTACHVGGGTATCTAATCC-3′, underline: overhang sequence for second-round PCR) over 20 cycles, and the PCR products were purified with Agencourt AMpure beads (Beckman Coulter) as described previously^15^. Then, the overhang and index sequences required for sequencing were added by a second round of PCR using NEBNext multiplex Oligos for Illumina (Dual Index Primers Set1, New England Biolabs) over eight cycles, and the products were purified with Agencourt AMpure beads. For each sample, equal amounts of each DNA amplicon library were mixed and sequenced on an MiSeq instrument (Illumina) using the MiSeq v3 Reagent kit and a 15% PhiX spike (Illumina). 16S rRNA gene analysis was performed using QIIME2 (v.2018.11; https://qiime2.org). In brief, raw sequence data were subjected to primer sequence trimming, quality filtering and paired-end read merging using the dada2 denoise-paired method (–p-trim-left-f 17 –p-trim-left-r 21–p-trunc-len-f 275 –p-trunc-len-r 215 –p-n-threads 16). Before taxonomic analysis, sequences of the 16S rRNA V3–V4 region were extracted from Greengenes 13_8 99% operational taxonomic units and our primer sequences using the q2-feature-classifier. Then, the Naive Bayes classifier was trained using the extracted Greengenes 13_8 reference sequences and Greengenes 13_8 99% operational taxonomic unit taxonomy. The taxonomic composition was visualized using a qiime taxa bar plot.

### Predicting *Enterococcus* domination

Predictive factors for *Enterococcus* domination, which was defined as a microbiota containing more than 25% of the genus *Enterococcus*, were examined using Cox proportional hazards regression. The first occurrence of *Enterococcus* domination in each case was defined as the end-point of interest. The following clinical variables were assessed as univariate predictors in each patient: age, sex, underlying diagnosis (acute leukaemia versus other diseases), conditioning regimen intensity (myeloablative conditioning versus reduced intensity conditioning), whether or not total body irradiation was included in the conditioning regimen, donor type (HLA-matched related donor versus HLA-matched unrelated donor, cord blood or haploidentical donor), graft type (bone marrow versus peripheral blood stem cell or cord blood) and antibiotic administration during the sample collection period (from the start of conditioning therapy to the final faecal sample collection in each case). The intensity of the conditioning regimen was defined according to a previous report from the American Society for Blood and Marrow Transplantation^62^. All statistical analyses were performed using EZR v.1.41 (Saitama Medical Center, Jichi Medical University), which is a graphical user interface for R^63^. *P* < 0.05 was considered significant.

### Isolation of intestinal bacteria

Enterococci were isolated by culturing faecal samples with *Enterococcus* domination on *Enterococcus* selective agar medium (brain–heart infusion (BHI) broth (BD) supplemented with 20 µg ml^−1^ aztreonam (Nacalai Tesque), 20 µg ml^−1^ polymyxin B (Nacalai Tesque), 4 µg ml^−1^ amphotericin B (Nacalai Tesque) and 50 µg ml^−1^ triphenyltetrazolium chloride (Nacalai Tesque)), or vancomycin-resistant *Enterococci*-selective agar (BD). After culturing at 37 °C aerobically for 24–48 h, colonies were picked and further cultured in BHI broth. After growth, the bacteria were stocked at −80 °C with 80% glycerol. DNA was extracted using the PowerSoil DNA Isolation Kit (Qiagen) in accordance with the manufacturer’s protocol. The species was determined for each strain by PCR using previously designed primers: 5′-ATCAAGTACAGTTAGTCT-3′ and 5′-ACGATTCAAAGCTAACTG-3′ for *E. faecalis* (product size: 941 bp), and 5′-TTGAGGCAGACCAGATTGACG-3′ and 5′-TATGACAGCGACTCCGATTCC-3′ for *E. faecium* (658 bp)^64,65^. PCR amplicons were analysed using MCE-202 MultiNA (Shimadzu).

*S. epidermidis*, *K. pneumoniae*, *K. oxytoca*, *C. freundii* and *E. coli* were isolated by culturing faecal samples with non-*Enterococcus* domination on Luria–Bertani (LB) agar medium (Nacalai Tesque). After culturing at 37 °C aerobically for 24–48 h, colonies were picked and further cultured in LB broth. After growth, the bacteria were stocked at −80 °C with 80% glycerol. DNA was extracted using the PowerSoil DNA Isolation Kit (Qiagen) in accordance with the manufacturer’s protocol. The species was determined for each strain by PCR and Sanger sequencing using previously designed primers: 5′-ACACGACGCTCTTCCGATCTCCTACGGGNGGCWGCAG-3′ and 5′-GACGTGTGCTCTTCCGATCTGACTACHVGGGTATCTAATCC-3′.

### Detection and quantification of cytolysin genes

Screening for cytolysin genes (*cylL*_*L*_, *cylM*, *cylB* and *cylA*) was performed by PCR using previously developed primers based on GenBank nucleotide sequences for the *E. faecalis* cytolysin operon (accession no. L37110): 5′-GATGGAGGGTAAGAATTATGG-3′ and 5′-GCTTCACCTCACTAAGTTTTATAG-3′ for *cylL*_*L*_ (product size: 253 bp), 5′-AAAAGGAGTGCTTACATGGAAGAT-3′ and 5′-CATAACCCACACCACTGATTCC-3′ for *cylM* (2,940 bp), 5′-AAGTACACTAGTAGAACTAAGGGA-3′ and 5′-ACAGTGAACGATATAACTCGCTATT-3′ for *cylB* (2,020 bp) and 5′-TAGCGAGTTATATCGTTCACTGTA-3′ and 5′-CTCACCTCTTTGTATTTAAGCATG-3′ for *cylA* (1,282 bp)^66^. PCR amplicons were analysed using MCE-202 MultiNA.

The quantity of *cylL*_*L*_ PCR amplicons was determined using MCE-202 MultiNA and *cylL*_*L*_ copy numbers were analysed. One nanogram of faecal DNA was used as template DNA.

### Haemolytic activity assays

Culture medium of each *E. faecalis* or *E. faecium* strain was streaked on Columbia agar plates supplemented with 5% horse blood (Nissui Pharmaceutical Co.). After aerobic incubation at 37 °C for 72 h, the presence of zones of clearing around the colonies was determined to indicate beta-haemolysis.

### Antimicrobial susceptibility testing

A suspension of each isolated *E. faecalis* or *E. faecium* strain prepared to 1 McFarland standard with physiological saline was diluted 500 times in BHI broth, and then cultured aerobically in the solid-phase wells of an Eiken DP42 dry plate (Eiken Chemical Co.) for 24 h. The minimum inhibitory concentrations were determined according to the manufacturer’s instructions.

### Whole-genome sequencing

#### Bacterial DNA sequencing

DNA libraries were prepared with the KAPA HyperPlus kit (KAPA Biosystems) following the manufacturer’s instructions, except that NEBNext multiplex Oligos were used for Illumina (New England Biolabs) at the adapter ligation and barcoding steps. The prepared target library size was 450–550 nucleotides. The concentration of the library was quantified with the KAPA Illumina Library Quantification kit (KAPA Biosystems) and adjusted to the mean library size measured by MCE-202 MultiNA. The libraries were pooled and sequenced on a HiSeq2500 sequencer (2 × 250 paired-end reads, HiSeq Rapid SBS Kit v2; Illumina). For each run, 10 libraries for the bacterial fractions were pooled at equimolar concentrations.

#### Processing of sequencing data

Sequencing reads were demultiplexed using Illumina CASAVA software and were processed with the following three steps: (1) adaptor sequence trimming; (2) nucleotide trimming and removal of duplicates; and (3) error base correction. First, adaptor sequences were removed by cutadapt software (http://cutadapt.readthedocs.io/en/stable/index.html) (v.1.18). Second, the first and last 10 nucleotides of each read were removed, then within 20 nucleotides from both ends, low-quality nucleotides with a Phred quality score of less than 20 were trimmed, and polynucleotides at the end of the sequence were also trimmed. After trimming, sequences shorter than 75 nucleotides, low-complexity sequences (DUST score greater than 7), exact duplicates, sequences containing N and singletons were filtered out. This step was performed by PRINSEQ software (http://prinseq.sourceforge.net/) (lite v.0.20.4) (-trim_right 10 -trim_left 10 -trim_qual_right 20 -trim_qual_left 20 -trim_qual_window 20 -trim_ns_right 1 -min_len 75 -lc_method dust -lc_threshold 7-ns_max_n 0 -derep 1). Third, correction of sequencing errors on the basis of the Hamming graph and Bayesian subclustering were performed using BayesHammer software (as bundled with SPAdes v.3.13.0) (spades.py –only-error-correction)^67^.

#### Metagenome assembly

The quality-filtered and error-corrected reads in each sample were assembled with MetaSPAdes v.3.13.0 with default k-mer lengths (options: --meta --only-assembler)^68^. To compare the abundance of contigs across the samples, the assembled contigs (with lengths ≥ 5 kb) from individual samples were pooled. CD-HIT-EST (v.4.8) was used to cluster pooled contigs at a 95% global average nucleotide identity (-c 0.95 -G 1 -n 10 -mask NX)^69^. From the non-redundant pooled contigs, circular contigs were identified by detecting overlaps in the 5′ and 3′ end sequences (more than 50 nucleotides overlap at 100% identity) of the contigs using Megablast (BLAST+ v.2.5)^70^. Each of the detected circular contigs was trimmed to remove redundant parts. Circular contigs longer than 1.5 kb and linear contigs longer than 5 kb were used for the analyses.

#### Bacterial taxonomic assignment

We assigned bacterial taxonomy to the contigs using PhyloPythiaS+ (v.1.4)^71^. The whole PhyloPythiaS+ pipeline was run (options: -n -g -o s16 mg -t -p c -r -s) using their reference database ‘NCBI201502’ with the configuration parameters: maxLeafllClades = 500 and minPercentInLeaf = 0.05 (the other parameters were set as default). To obtain the taxonomic profile in each sample, the quality-filtered and error-corrected reads were mapped to microbial taxonomy-specific marker genes with MetaPhlAn2.0 (v.2.5.0)^72^.

#### Read coverage calculation of contigs

The quality-filtered and error-corrected reads were mapped to the non-redundant pooled contigs using the bbmap (v.38.76) tool from BBtools (v.37.68) with at least 95% identity and the ambiguous mapping option (ambiguous = random). A contig was considered as ‘detected’ in a sample if more than 75% of the contig length was covered by mapped reads, as recommended in a previous study^73^. The abundance of a contig was calculated as the average contig coverage (number of nucleotides mapped to the contig divided by the contig length), where the abundance of a non ‘detected’ contig was set to 0, and normalized by the total number of nucleotides of the mapped reads in a sample, to obtain a total number of nucleotides equal to 10^9^.

#### Statistical analysis of gene functions

The detected ORFs in each sample were annotated according to KEGG (16 September 2018) prokaryote genes and corresponding KOs using the most significant hit (*E* value < 1 × 10^−5^ and bitscore > 50). The abundance of each KO term was calculated as the sum of per base read depths of the ORFs assigned to the KO divided by the sum of the lengths of these ORFs. The read depths were counted by samtools bedcov. We calculated the median abundances of 31 KOs considered to be the single copy genes (K01889, K01890, K02528, K02600, K02863, K02864, K02867, K02871, K02874, K02876, K02881, K02886, K02890, K02892, K02895, K02906, K02931, K02933, K02946, K02948, K02950, K02952, K02956, K02961, K02965, K02967, K02982, K02988, K02992, K02994 and K02996) for each sample and performed normalization by dividing the abundances of each KO by the median abundance of this set. Samples with a median abundance of zero were discarded from the analysis. The Wilcoxon rank-sum test was performed using the R packages coin, exactRankTests and qvalue. A volcano plot was drawn using the ggplot2 (v.3.3.6) R package.

#### Analysis of prophages

Prophage sequences in the bacterial contigs were predicted according to known viral signatures with VirSorter (v.1.0.3)^74^. The bacterial contigs (≥5 kb) from individual samples were analysed by VirSorter using both RefSeqABVir (–db 1) and Viromes (–db 2). To remove virus-derived sequences from the bacterial contigs, predicted prophage sequences of VirSorter categories 4 or 5 (presence of viral hallmark genes or enrichment of viral-like genes in a prophage region) were extracted. The positions of the predicted prophage sequences on the non-redundant pooled bacterial contigs were obtained through Megablast (BLAST+ v.2.5) searches (*E* value < 10^−100^ and at least 95% identity), and prophage sequences were merged if their positions overlapped. Finally, prophage sequences longer than 3 kb were extracted and listed as candidate prophage sequences.

#### Viral nucleotide and protein database

To classify the prophage contigs, we prepared viral genome and protein databases. The viral RefSeq sequences (released as of 5 May 2020; 366,089 proteins) were downloaded from the NCBI FTP site (https://ftp.ncbi.nlm.nih.gov/refseq/release/viral/). Taxonomic lineage information was assigned against NCBI RefSeq nucleotide sequence data, which were searched using the term “taxid = 28883 (Caudovirales)” on the NCBI website (https://www.ncbi.nlm.nih.gov/nuccore/) with 57,556 nucleotide hits, and then all sequences were downloaded as fasta files on 24 October 2021. The ORFs for the rest of the viral contigs were predicted for classification as described below.

#### Viral classification

We first classified prophage contigs using viral RefSeq genomes. The prophage contig sequences were searched against the viral RefSeq data mentioned above (‘Viral nucleotide and protein database’) using blastn (BLAST+ v.2.5). The results were sorted by value (minimum *E* value, maximum bitscore and maximum Query Cover), and the top three results were filtered. Because some viral contig sequences contained multiple fragments that were mapped to different viral genomes, the top three significant alignments were considered to determine viral taxonomy. If the top three alignments covered more than 50% of a contig sequence, the lowest common ancestor of the top three hits was determined using blast2lca (v.0.800) (https://github.com/emepyc/Blast2lca) (modified to use accession version identifiers) with the NCBI taxonomy (downloaded on 30 November 2017), and was assigned to the contig.

#### Gene prediction

The ORFs on the contigs were predicted using MetaProdigal (v.2.6.3) with the metagenomics procedure (-p meta)^75^. To predict genes spanning the 3′ to 5′ ends of a circular contig, a temporary version of the circular contig was used in the ORF prediction, in which the first 1,500 nucleotides were duplicated and added at the end of the contig.

#### ORF classification

To annotate the predicted ORFs, the amino acid sequences of the ORFs were queried by GHOST-MP (v.1.3.4) against the RefSeq viral protein (released as of 5 May 2020) with an *E* value < 10^−30^ and a bitscore > 50 (ref. ^76^). The viral RefSeq proteins with the top three closest homologies (*E* value < 10^−5^ and bitscore > 50) were considered for each ORF. With regard to the prophage region, the ORFs were classified according to their PHROG category^77^. The PHROG database (v.4) was downloaded from https://phrogs.lmge.uca.fr/ as fasta files, then converted into a Blast database. The prophage ORFs were annotated using blastp, then the annotated ORFs were described using Circos^78^ and coloured according to their PHROG category.

#### Analysis of phage-derived endolysins

The ORFs that putatively encode endolysin proteins were extracted from the prophage regions found in the 11 *E. faecalis* strains using their ORF annotations. The viral RefSeq protein annotations containing “Endolysin”/“endolysin” were extracted using blastp (BLAST+ v.2.5) and searched against the RefSeq bacterial protein database downloaded from the NCBI ftp site (https://ftp.ncbi.nlm.nih.gov/refseq/release/bacteria/) on 17 January 2021, including non-redundant proteins. To avoid false positives, the ORFs were annotated with a significant *E* value < 10^−30^. Multiple alignments of the protein sequences in each group were performed with MUSCLE (v.3.8.31) with default settings^79^. Jalview (v.2.11.3.2) was used to visualize the multiple alignment results^80^. To find the domains of the detected proteins, we searched the accession number on the NCBI website (https://www.ncbi.nlm.nih.gov/protein/) on 25 January 2022.

Gene prediction was performed as described above and each gene is shown in Extended Data Fig. 2. His-SUMO-tagged endolysins were obtained by PCR using genomic DNA from the *E. faecalis* strain obtained from Patient031_14_8 as a template and were ligated into the BamHI and SalI sites of the pCold-SUMO expression vector (Creative Biogene)^81^. The primer sequences were as follows: forward primer, 5′-AAGGATCCATGACATTAAACGGAATTGA-3′ and reverse primer, 5′-AAGTCGACTTACCCATAGATTAATTTCT-3′. The plasmids were then transformed into BL21 (DE3) cells, which were grown in LB medium containing 100 µg ml^−1^ ampicillin at 37 °C until the optical density at 600 nm (OD_600 nm_) reached 0.4–0.6. Isopropyl β-d-thiogalactoside (IPTG) (Nacalai Tesque) was added until the final concentration reached 1 mM, and the culture was incubated at 16 °C for 18 h at 120 rpm. After centrifugation at 3,000*g* for 15 min, the pellet was washed with sterile deionized water. After centrifugation at 3,000*g* for 15 min, the precipitate was resuspended in xTractor buffer (TaKaRa). The lysate was incubated with 10 U ml^−1^ of DNase I (Roche) and 100 mg ml^−1^ of hLysozyme (Sigma-Aldrich) for 30 min, and disrupted by sonication (20 s pulse, 80 s rest, over 10 min). After centrifugation at 12,000*g* for 30 min, the supernatant was sterile-filtered through 0.45- and 0.2-µm filters. The target proteins were purified through Capturem His-Tagged Purification Maxiprep columns (TaKaRa). The lysate was loaded onto the column, which was equilibrated with xTractor buffer, and then centrifuged at 2,000*g* for 3 min at room temperature. The column was washed with wash buffer (20 mM Na_3_PO_4_ (Nacalai Tesque), 150 mM NaCl, pH 7.6), and the target protein was eluted with elution buffer (20 mM Na_3_PO_4_, 500 mM NaCl, 500 mM imidazole, pH 7.6 (Nacalai Tesque)).

All samples were desalted by HiTrap desalting columns (Cytiva) with HiTrap buffer (50 mM Na_3_PO_4_, 0.15 M NaCl, pH 7.0). The protein solution was loaded into the concentration tube (Amicon Ultra-15 10K; Merck) and centrifuged at 5,000*g* for 20 min at room temperature. The concentration of the target protein was measured using Protein Assay CBB Solution (Nacalai Tesque).

#### Western blotting

The samples collected were purified and concentrated as described above, then subjected to 5–20% SDS–PAGE. The resolved proteins were transferred to a polyvinyl difluoride membrane (Bio-Rad), which was incubated with anti-His antibody (1:2,000) (Proteintech). The membrane was washed three times with TBS-T and then incubated with goat anti-mouse IgG(H+L) (1:10,000) (Jackson Immunoresearch). Immunoreactivity was detected using SuperSignal (Thermo Fisher Scientific).

### Lytic assay against planktonic *E. faecalis* and *E. faecium*

The *E. faecalis* strain Patient031_14_8 and *E. faecium* strains Patient012_7_1, Patient012_7_2, Patient016_14_1, Patient016_14_2, Patient019_14_1, Patient038_35_1, Patient038_35_6, Patient040_35_1, Patient040_35_2, Patient040_42_1, Patient040_42_3, Patient046_14_1, Patient046_14_2, Patient046_21_1, Patient046_21_2, Patient050_7_3, Patient052_21_1, Patient052_21_2 and Patient052_28_1 were grown in BHI broth aerobically and then collected by centrifugation at 3,000*g* for 15 min. Cell pellets were washed and resuspended in HiTrap buffer. The lytic activity of the endolysin was calculated based on reduction at an OD_600 nm_ as measured in a TVS062CA BioPhoto recorder (ADVANTEC), with the OD_600 nm_ measured every minute. For each sample, a 50 µg ml^−1^ final concentration of endolysin or a 50 µg ml^−1^ final concentration of vehicle including His-SUMO was added to the cell resuspension.

### Lytic assay on *E. faecalis* biofilm

A biofilm assay using crystal violet staining was conducted according to previous studies^12,82,83^. In brief, each isolated *E. faecalis* strain was cultured overnight in BHI medium supplemented with 20 µg ml^−1^ aztreonam, 20 µg ml^−1^ polymyxin B and 4 µg ml^−1^ amphotericin B at 37 °C. A sample of each overnight culture was diluted 100-fold in fresh BHI medium, inoculated into a 96-well flat-bottomed polystyrene microtiter plate (Corning) and further incubated aerobically at 37 °C for 24 h. After incubation, the 96-well plates were gently washed once with 200 µl HiTrap buffer, and then 200 µl of endolysin or vehicle including His-SUMO (50 µg ml^−1^ final concentration) was added. The plates were incubated overnight, then washed once with 200 µl phosphate-buffered saline (PBS) to remove planktonic cells, and dried in an inverted position. Subsequently, 200 µl of 0.5% crystal violet solution was added for staining. After 15 min of staining at room temperature, the plates were washed three times with 200 µl of PBS to remove excess dye. The plates were dried at 50 °C for 30 min, and the bound dye was dissolved by adding 200 µl of 95% ethanol (v/v) for 20 min. The absorbance of the samples was measured at 570 nm. All experiments included eight replicate experimental wells.

### In vivo mouse models

Female germ-free C57BL/6 (H2kb) and female specific-pathogen-free 129SvJ/JmsSlc mice (6–8 weeks old) were purchased from SLC Japan and CLEA Japan, respectively. Mice were housed in a temperature-controlled (23 ± 2 °C) room with a dark period from 20:00 to 08:00. They were allowed free access to sterile water and standard laboratory mouse chow. Germ-free C57BL/6 mice were reared as one individual per cage. All mice were randomly housed in groups and selected for the experiments.

In the mono-colonized gnotobiotic mouse model, C57BL/6 germ-free female mice were orally inoculated with *E. faecalis* strain Patient031_14_8, *E. faecalis* strain Patient009_35_10, *E. faecalis* strain Patient015_56_7, *E. faecium* strain Patient038_35_1, *E. faecium* strain Patient040_35_1, *E. faecalis* strain JCM5803, *E. faecium* strain Patient019_14_1 or *E. coli* strain Patient025_0_122 suspensions (2.0 × 10^8^ CFU freshly suspended in 200 µl of sterile PBS per mouse) before stem cell transplantation. The *E. faecalis* strain JCM5803 was obtained from the Japan Collection of Microorganisms. Three weeks after the transfer, bacterial colonization was confirmed by culturing faecal suspensions (diluted in PBS with vortex mixing, and then passed through a 100-µm cell strainer) on *Enterococcus* selection agar medium (BHI broth containing 20 µg ml^−1^ aztreonam, 20 µg ml^−1^ polymyxin B, 4 µg ml^−1^ amphotericin B and 50 µg ml^−1^ triphenyltetrazolium chloride) or LB agar medium.

In a humanized gnotobiotic mouse model, 200-µl suspensions of Patient031_14 faecal samples (samples obtained from Patient031 on day 14 ± 3), Patient043_42 faecal samples (samples obtained from Patient043 on day 42 ± 3), Patient032_21 faecal samples (samples obtained from Patient032 on day 21 ± 3), Patient026_7 faecal samples (samples obtained from Patient026 on day 7 ± 3) or Patient062_0 faecal samples (samples obtained from Patient062 on day 0 ± 3) in anaerobic culture medium at a 16-fold dilution (w/v) were administered before stem cell transplantation. Three weeks after the transfer, the intestinal bacterial composition of each mouse was analysed by 16S rRNA gene sequencing.

In the humanized GVHD model colonized with Patient031_14 faecal samples, Patient043_42 faecal samples or Patient032_21 faecal samples, germ-free mice were reared in two or more germ-free isolators, and each isolator contained mice from the endolysin-treated group and the vehicle-treated group. All animal experiments were performed with the approval of the Animal Care and Use Committees of Osaka Metropolitan University.

#### Lytic assay in the gnotobiotic mouse model

Two hundred micrograms of endolysin or vehicle, dissolved in 200 µl of HiTrap buffer, was orally administered three times a week to *E. faecalis*-mono-colonized gnotobiotic mice (*n* = 2 per group). After a week of administration, mice were euthanized and the entire intestinal tract was immediately removed and divided into the ileum and colon. Intestinal tract samples were also obtained from a germ-free mouse as control samples. Specimens of approximately 3 mm^3^ were prepared and then fixed in 2% formaldehyde and 2.5% glutaraldehyde in 0.1 M phosphate buffer. After extensive washing, secondary fixation in 1% osmium tetroxide solution in 0.1 M phosphate buffer was performed for 2 h, followed by further washing and chemical dehydration in a progressive ethanol series. Samples were transferred to isoamyl acetate for 30 min, and then dehydrated in a Hitachi Model HCP-2 critical point dryer (Hitachi) with liquid CO_2_. Samples were finally coated with 5 nm of osmium in an Neoc-Pro/PN (Meiwafosis) before imaging using a Hitachi S-4700 (Hitachi).

Two hundred micrograms of endolysin or vehicle dissolved in 200 µl of HiTrap buffer was orally administered three times a week to *E. faecium*-mono-colonized gnotobiotic mice (*n* = 4 per group). After a week of administration, the CFU was determined by culturing faecal suspensions (diluted in PBS with vortex mixing, and then passed through a 100 µm cell strainer) on *Enterococcus* selection agar medium (BHI broth containing 20 µg ml^−1^ aztreonam, 20 µg ml^−1^ polymyxin B, 4 µg ml^−1^ amphotericin B and 50 µg ml^−1^ triphenyltetrazolium chloride).

#### Gnotobiotic GVHD mouse model

A previously reported aGVHD mouse model based on chemotherapy conditioning and major-histocompatibility-complex-matched (minor-antigen-mismatched) transplantation was used in our study^84^.

As conditioning therapy, female germ-free C57BL/6 mice received intraperitoneal doses of busulfan (Sigma-Aldrich; 20 mg per kg per day) for five days, followed by cyclophosphamide (Sigma-Aldrich; 100 mg per kg per day) for three days. Day −2 and −1 were rest days. On day 0, recipient C57BL/6 mice were intravenously injected with 1.5 × 10^7^ bone marrow cells and 2.0 × 10^6^ splenic T cells from female 129SvJ/JmsSlc donor mice. Bone marrow cells were flushed from the femur and suspended in Hanks’ balanced salt solution (Nacalai Tesque). After erythrocyte lysis using Red Blood Cell Lysing Buffer Hybti-Max (Sigma-Aldrich), a single-cell suspension was prepared in PBS. A splenic T cell suspension was obtained using the Pan T Cell Isolation Kit II for mice (Milltenyi Biotec) according to the manufacturer’s instructions.

From the day of stem cell transplantation, 200 µg of endolysin or vehicle, dissolved in 200 µl HiTrap buffer, was administered orally three times a week for three weeks. Survival was monitored daily. Overall survival in each group was statistically analysed and compared using a generalized Wilcoxon test. Using the *E. faecalis*-mono-colonized gnotobiotic GVHD mouse model, faecal *E. faecalis* bacteria were quantified by culturing faecal suspensions diluted in BHI broth on *Enterococcus* selection agar medium and counting the colonies. All experiments were performed in duplicate. Using the humanized gnotobiotic GVHD mouse model, bacterial DNA was extracted from sequentially collected faecal samples using the PowerSoil DNA Isolation Kit (Qiagen) in accordance with the manufacturer’s protocol and 16S rRNA gene analysis was performed as described above. Faith’s phylogenetic alpha diversity estimate and principal coordinate analysis of the weighted UniFrac distance matrices were performed using QIIME2 (v.2018.11; https://qiime2.org).

The concentration of IFNγ in the serum on day 8 after the transfer was measured by ELISA using reagents from Invitrogen, according to the manufacturer’s protocol.

### Reporting summary

Further information on research design is available in the Nature Portfolio Reporting Summary linked to this article.

## Online content

Any methods, additional references, Nature Portfolio reporting summaries, source data, extended data, supplementary information, acknowledgements, peer review information; details of author contributions and competing interests; and statements of data and code availability are available at 10.1038/s41586-024-07667-8.

## Supplementary information

Supplementary Information

Reporting Summary

## Source data

Source Data Fig. 1

Source Data Fig. 2

Source Data Fig. 3

Source Data Fig. 4

Source Data Extended Data Fig. 1

Source Data Extended Data Fig. 3

Source Data Extended Data Fig. 4

Source Data Extended Data Fig. 5

Source Data Extended Data Fig. 6

Source Data Extended Data Fig. 7

Source Data Extended Data Fig. 8

## Figures and Tables

**Extended Data Table 1:**
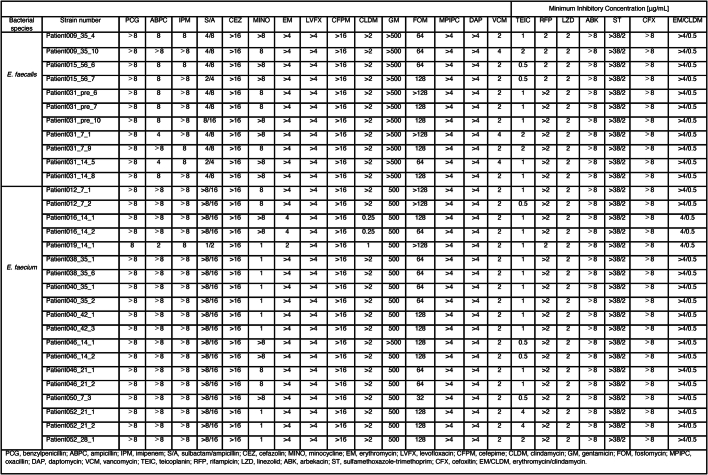
Antimicrobial susceptibility testing of *Enterococcus* species isolated from patients with allo-HCT

Antimicrobial susceptibility testing for the 11 isolated *E. faecalis* and the 19 isolated *E. faecium* was performed and the minimum inhibitory concentrations were determined.

## Data Availability

All data supporting the findings of this study are provided within the manuscript and its Supplementary Information. Sequencing data generated for this study have been deposited in the NCBI Sequence Read Archive (PRJNA1095194 and PRJNA1109929). The RefSeq protein database downloaded from the NCBI ftp site on 5 May 2020 and 17 January 2021, and the NCBI RefSeq nucleotide sequence data downloaded on 24 October 2021, have been deposited in the Zenodo repository (10.5281/zenodo.11196056 (ref. ^85^) and 10.5281/zenodo.11239382 (ref. ^86^)). Source data are provided with this paper.

## Code availability

Additional scripts are available in the Zenodo repository at 10.5281/zenodo.11401108 (ref. ^87^).
