# Supplementary material for: An enterococcal phage-derived enzyme suppresses graft-versus-host disease
Source: Nature. 2024 Jul 10;632(8023):174–81. doi: 10.1038/s41586-024-07667-8 (PMC11291292; doi:10.1038/s41586-024-07667-8)
Supplement: Supplementary file 1 — Supplementary Information [file 41586_2024_7667_MOESM1_ESM.pdf]

nature portfolio

https://doi.org/10.1038/s41586-024-07667-8

Supplementary information

An enterococcal phage-derived enzyme suppresses graft-versus-host disease

In the format provided by the authors and unedited

Supplementary Information for

An enterococcal phage-derived enzyme suppresses graft-versus-host disease

Authors

Kosuke Fujimoto[1,2,*], Tetsuya Hayashi[1,3,*], Mako Yamamoto[4], Noriaki Sato[4], Masaki Shimohigoshi[1], Daichi Miyaoka[1], Chieko Yokota[1], Miki Watanabe[1], Yuki Hisaki[1], Yukari Kamei[1], Yuki Yokoyama[1], Takato Yabuno[1], Asao Hirose[3], Mika Nakamae[3,5], Hirohisa Nakamae[3], Miho Uematsu[1], Shintaro Sato[1,6], Kiyoshi Yamaguchi[7], Yoichi Furukawa[7], Yukihiro Akeda[8], Masayuki Hino[3,5], Seiya Imoto[4,9], and Satoshi Uematsu[1,2,9,10,11]

Affiliations

[1]Department of Immunology and Genomics, Graduate School of Medicine, Osaka Metropolitan University, Abeno-ku, Osaka, Japan

[2]Division of Metagenome Medicine, Human Genome Center, The Institute of Medical Science, The University of Tokyo, Minato-ku, Tokyo, Japan

[3]Hematology, Graduate School of Medicine, Osaka Metropolitan University, Abeno-ku, Osaka, Japan

[4]Division of Health Medical Intelligence, Human Genome Center, The Institute of Medical Science, The University of Tokyo, Minato-ku, Tokyo, Japan

[5]Department of Laboratory Medicine and Medical Informatics, Graduate School of Medicine, Osaka Metropolitan University, Osaka, Japan

[6]Department of Microbiology and Immunology, School of Pharmaceutical Sciences, Wakayama Medical University, Wakayama, Japan

[7]Division of Clinical Genome Research, The Institute of Medical Science, The University of Tokyo, Tokyo, Japan

[8]Department of Bacteriology I, National Institute of Infectious Diseases, Tokyo, Japan

[9]Collaborative Research Institute for Innovative Microbiology, The University of Tokyo, Bunkyo-ku, Tokyo, Japan

[10]Reseach Institute for Drug Discovery Science, Osaka Metropolitan University, Abeno-ku, Osaka, Japan

[11]International Research Center for Infectious Diseases, Osaka Metropolitan University, Abeno-ku, Osaka, Japan

[*]These authors contributed equally to this work

Correspondence to:

Satoshi Uematsu

Department of Immunology and Genomics, Graduate School of Medicine, Osaka Metropolitan University, Abeno-ku, Osaka 545-8585, Japan

Tel: +81-6-6645-3926

Fax: +81-6-6645-3927

E-mail: uematsu.satoshi@omu.ac.jp

Division of Metagenome Medicine

Human Genome Center

The Institute for Medical Science, The University of Tokyo

4-6-1 Shirokanedai, Minato-ku, Tokyo 108-8639, Japan

Tel: +81-3-6409-2163

Fax: +81-3-6409-2391

Seiya Imoto

Division of Health Medical Intelligence, Human Genome Center, The Institute of Medical Science, The University of Tokyo, 4-6-1 Shirokanedai, Minato-ku, Tokyo 108-8639, Japan

Tel.: +81-3-5449-5615

Fax: +81-3-5449-5442

E-mail: imoto@ims.u-tokyo.ac.jp

Table of Contents

Supplementary Fig. 1. Raw images related to Figure 1a

Supplementary Fig. 2. Multiple alignment of the detected *E. faecalis* endolysins and WP_002399372 sequences

Supplementary Fig. 3. Raw image related to Figure 2c

Supplementary Fig. 4. Raw image related to Figure 2d

Supplementary Fig. 5. Raw image related to Figure 2f

Supplementary Fig. 6. Schematic diagrams of gnotobiotic GVHD mice related to Figure 3 and Figure 4

Supplementary Table 1. Patient characteristics

Supplementary Table 2. Detailed patient characteristics

Supplementary Table 3. Predictive factors of *Enterococcus* domination

Supplementary Figure 1

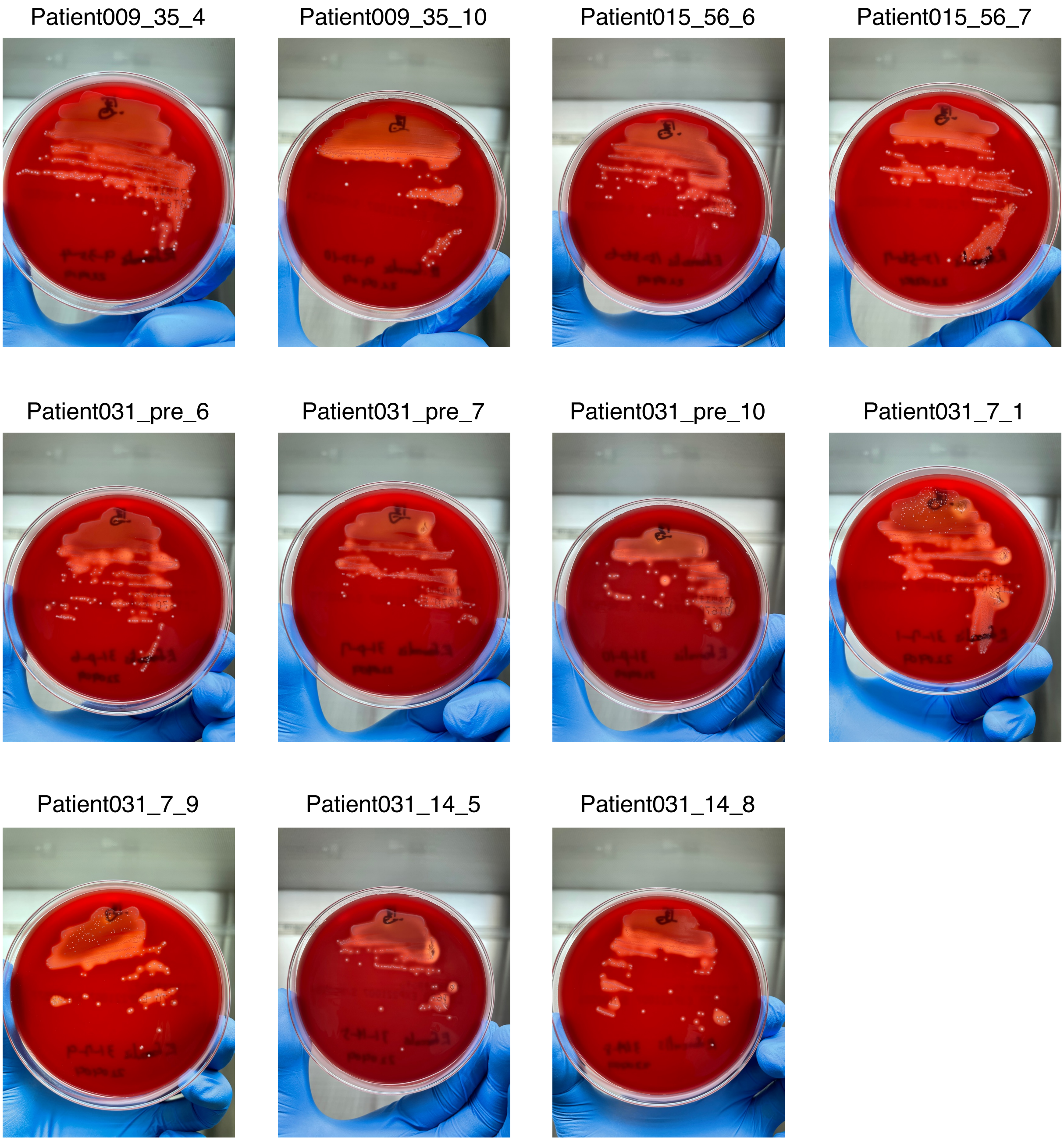

Patient012_7_1

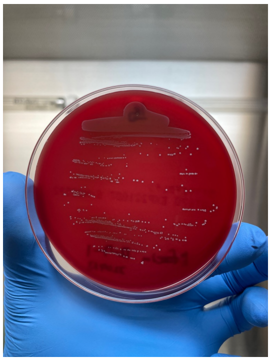

Patient012_7_2

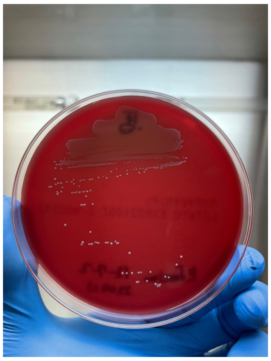

Patient016_14_1

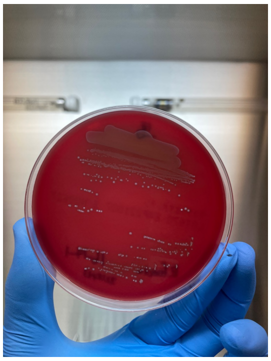

Patient016_14_2

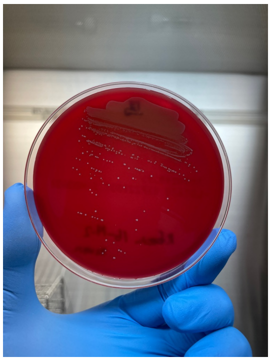

Patient019_14_1

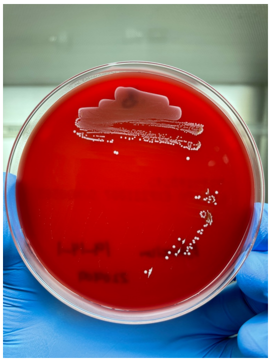

Patient038_35_1

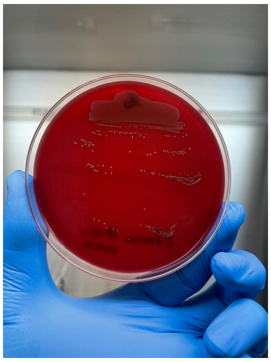

Patient038_35_6

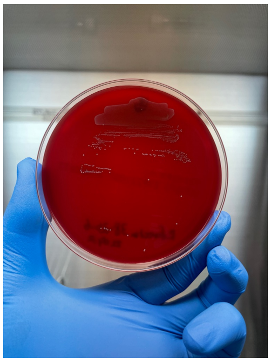

Patient040_35_1

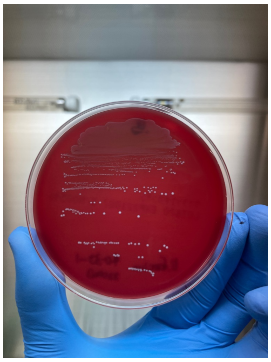

Patient040_35_2

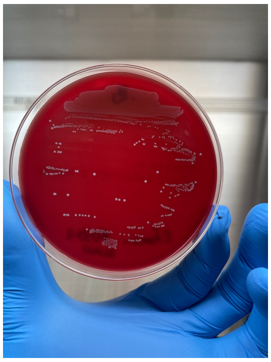

Patient040_42_1

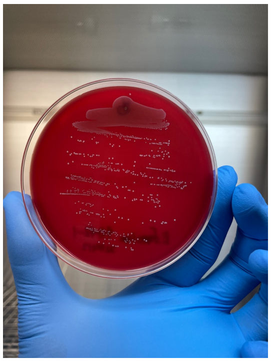

Patient040_42_3

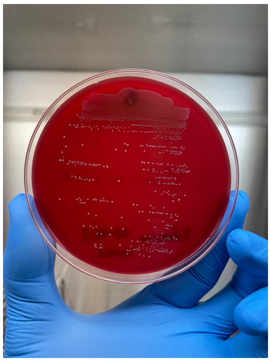

Patient046_14_1

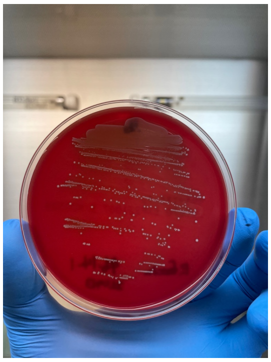

Patient046_14_2

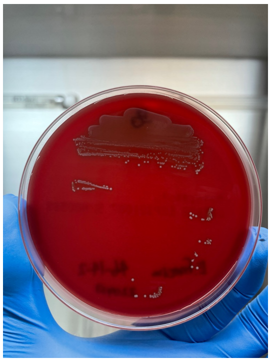

Patient046_21_1

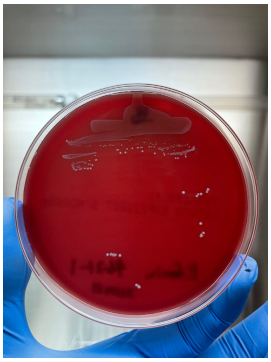

Patient046_21_2

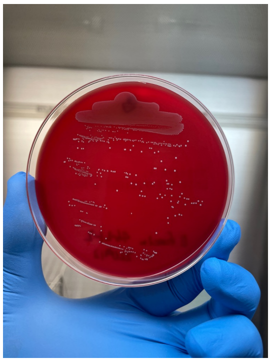

Patient050_7_3

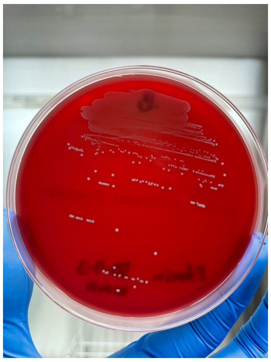

Patient052_21_1

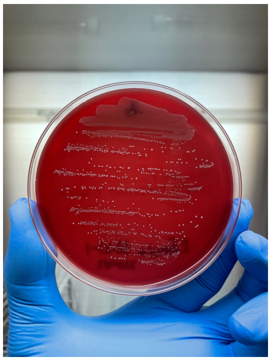

Patient052_21_2

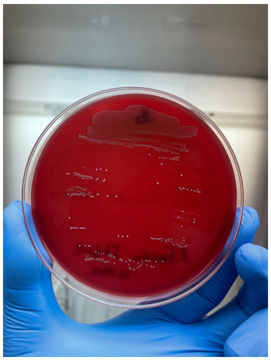

Patient052_28_1

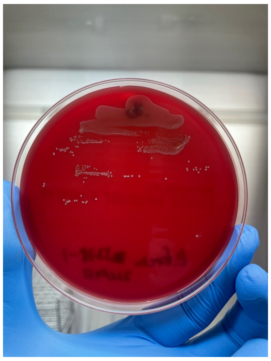

Supplementary Fig. 1: Raw images related to Figure 1a.
Detection of functional haemolytic activity. A representative image is shown.

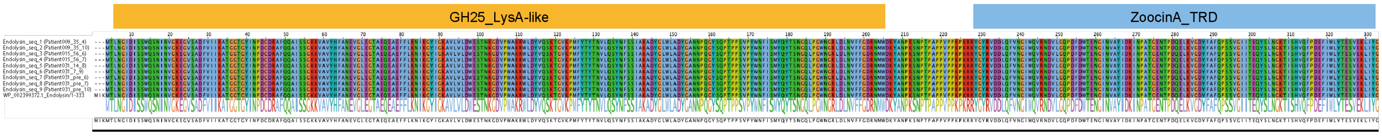

Supplementary Fig. 2: Multiple alignment of the detected *E. faecalis* endolysins and WP_002399372 sequences

The aligned amino acid sequences (top) and their conservation scores (middle) and consensus sequences (bottom) are shown.

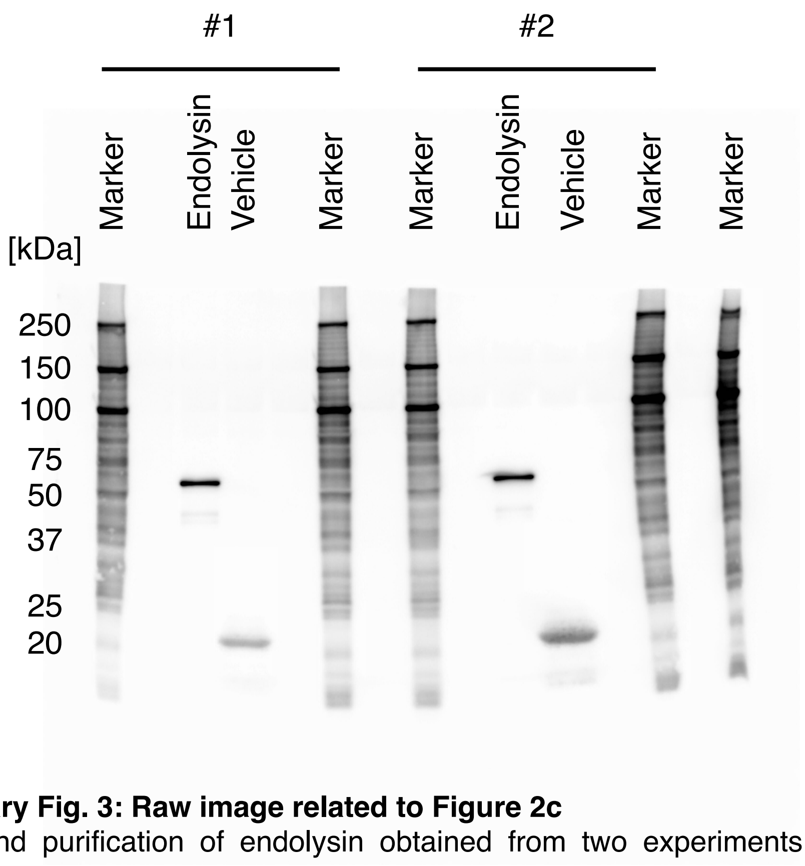

Supplementary Fig. 3: Raw image related to Figure 2c

Expression and purification of endolysin obtained from two experiments (#1 and #2) by western blotting. A representative blot is shown.

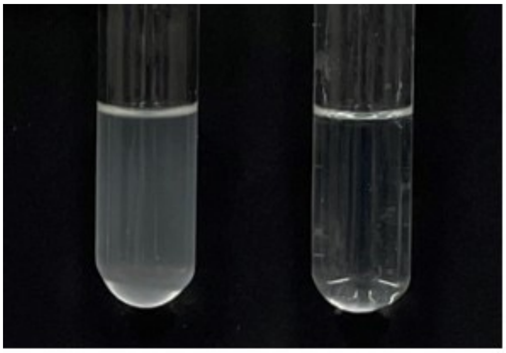

Supplementary Fig. 4: Raw image related to Figure 2d
Bacteriolytic capacity of endolysins against an *E. faecalis* strain obtained from Patient031_14_8. A representative image is shown.

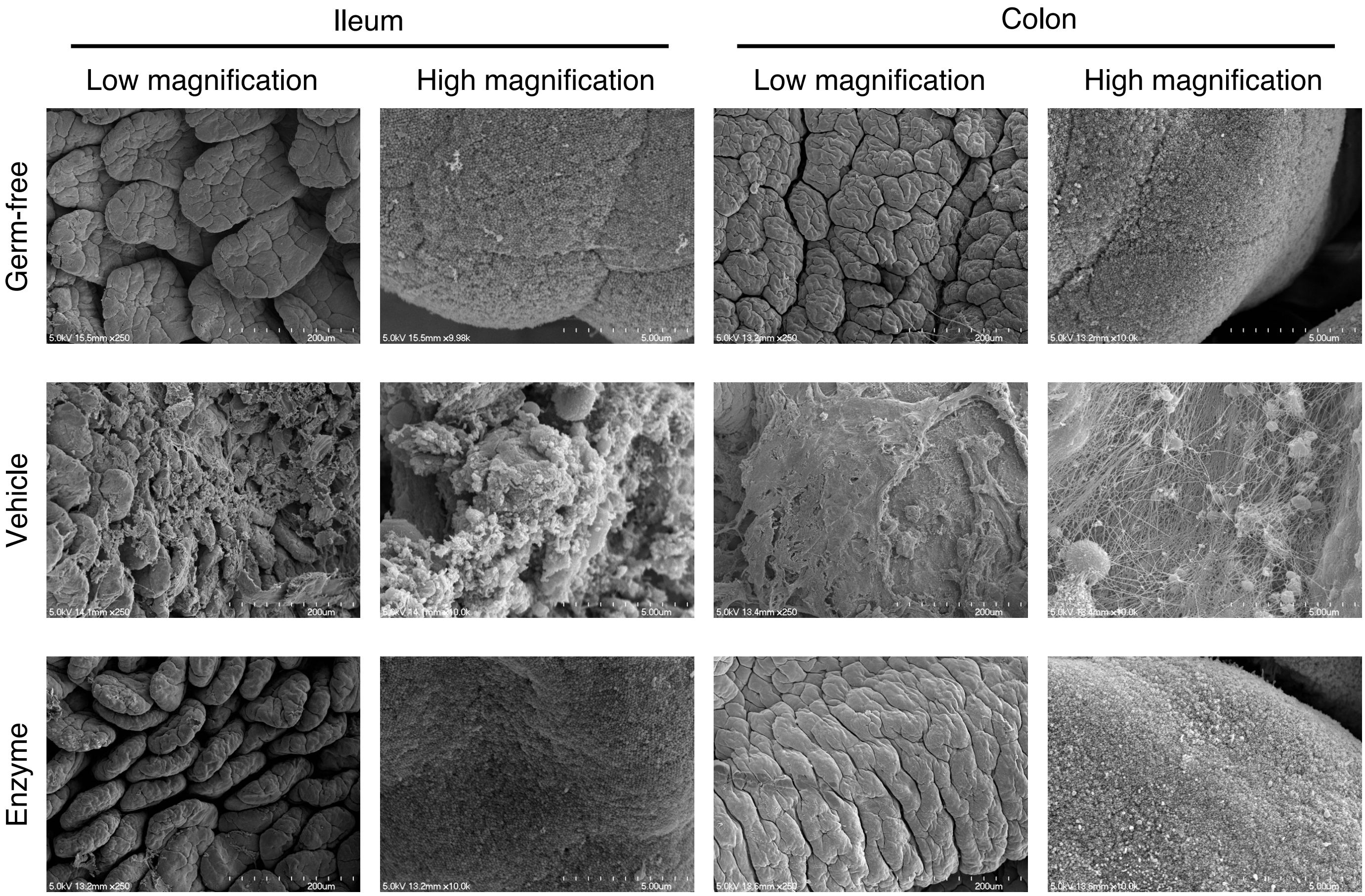

Supplementary Fig. 5: Raw images related to Figure 2f

Oral administration of endolysin or vehicle in germ-free mice or gnotobiotic mice mono-colonized with *E. faecalis*. Representative scanning electron microscopic images of the small intestines and large intestines are shown.

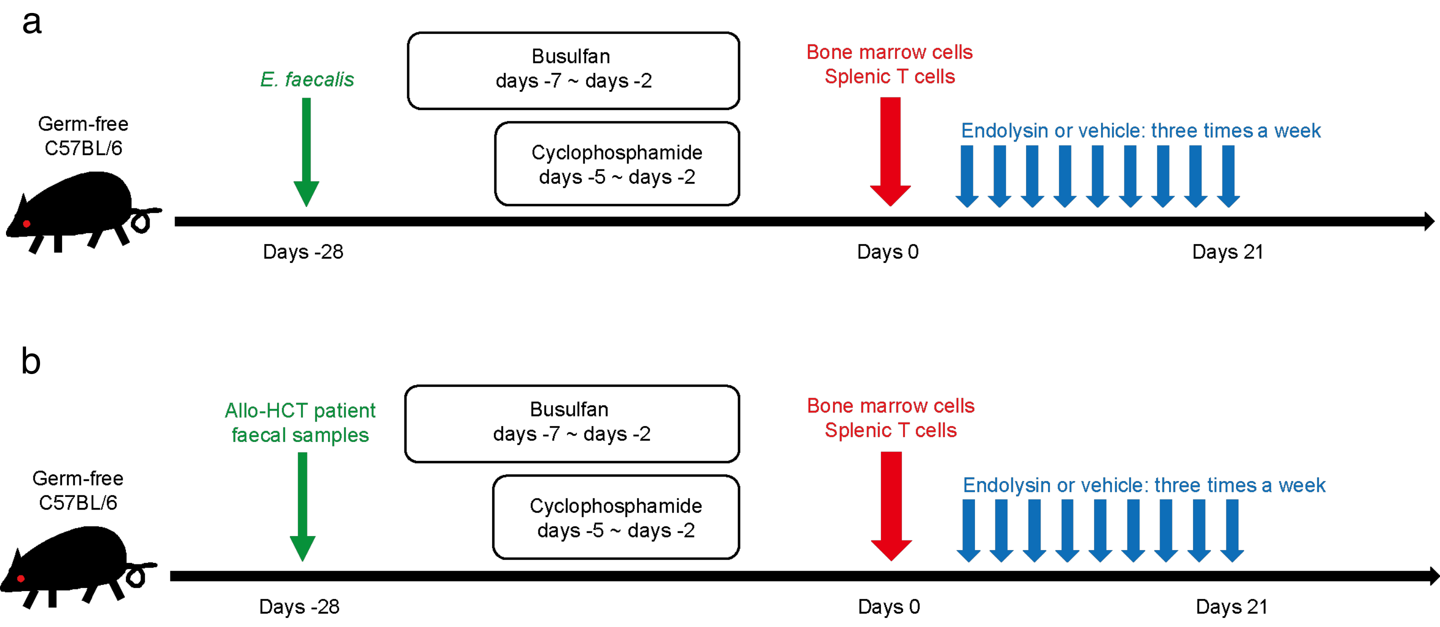

Supplementary Fig. 6: Schematic diagrams of gnotobiotic GVHD mice

a, Schematic diagram of *E. faecalis* mono-colonized gnotobiotic GVHD mice.

b, Schematic diagram of humanized gnotobiotic GVHD mice inoculated with the human faecal microbiota of an allo-HCT patient.

Supplementary Table 1. Patient characteristics

	***n* = 46**
**Age (median, range)**	54.5 (19–72)
**Sex (male) [no. (%)]**	25 (54.3)
**Disease [no. (%)]**	
acute leukaemia	26 (56.5)
myelodysplastic syndrome/myeloproliferative neoplasms	12 (26.1)
malignant lymphoma	7 (15.2)
other disease	1 (2.2)
**Donor source [no. (%)]**	
matched related donor	10 (21.7)
matched unrelated donor	7 (15.2)
mismatched unrelated donor	1 (2.2)
cord blood	11 (23.9)
haploidentical donor	17 (37.0)
**Graft type [no. (%)]**	
bone marrow	8 (17.4)
peripheral blood stem cells	27 (58.7)
cord blood	11 (23.9)
**Conditioning regimen [no. (%)]**	
myeloablative conditioning	24 (52.2)
reduced intensity conditioning	21 (45.7)
nonmyeloablative conditioning	1 (2.2)
**Total body irradiation-containing regimen [no. (%)]**	
yes	14 (30.4)
no	32 (69.6)
**Antibiotic administration* [no. (%)]**	
**Prophylactic antibiotics**	
fluoroquinolone	44 (95.7)
sulfamethoxazole-trimethoprim	39 (84.5)
isoniazid	3 (6.5)
macrolide	2 (4.3)
**Empiric or targeted antibiotics**	
cefems (cefepim or cefozopran)	21 (45.7)
piperacillin/tazobactam	26 (56.5)
carbapenems (meropenem or doripenem)	17 (37.0)
glycopeptides (vancomycin or teicoplanin) or lipopeptides (daptomycin)	28 (60.9)
metronidazole	3 (6.5)

* Antibiotics administered during the sample collection periods (from the start of conditioning therapy to the final faecal sample collection in each case) are listed. Antibiotic administrations are not mutually exclusive and do not total 100%.

Supplementary Table 2. Detailed patient characteristics

Patient number*	Age/Sex	Disease	Donor	Graft type	Conditioning therapy		Acute GVHD								With *Enterococcus* domination	Cytolysin positivity
					Intensity	TBI containing	Max grade	Onset	Max	Organ involvement (Max stage)			Treatment	Response		
										Skin	Liver	Gut				
001	59/M	Lymphoma	MRD	BM	MAC	No									Yes	No
002	61/M	MDS/MPN	Haplo	PB	RIC	No	II	day 2	day 17	3	0	0	mPSL[†]	CR	No	
004	60/F	AL	CB[‡]	CB	RIC	Yes									Yes	No
005	47/F	AL	CB	CB	MAC	No	III	day 13	day 13	1	0	2	mPSL	CR	Yes	Yes
006	19/F	AL	Haplo	PB	RIC	No	I	day 21	day 21	2	0	0	topical steroid	CR	No	
007	42/F	AL	MRD	BM	MAC	Yes									Yes	No
008	47/M	AL	Haplo	PB	RIC	No									Yes	No
009	42/M	AL	MRD	PB	MAC	No	II	day 14	day 16	0	0	1	mPSL	CR	Yes	Yes
010	37/M	Lymphoma	CB	CB	RIC	Yes	III	day 6	day 6	0	0	2	mPSL	CR	No	
011	62/M	MDS/MPN	Haplo	PB	RIC	No									Yes	No
012	30/F	AL	MRD	PB	MAC	No									Yes	No
013	42/F	AL	MRD	PB	NMA	Yes									No	
015	38/M	MDS/MPN	MRD	PB	MAC	No	III	day 33	day 34	0	0	2	mPSL	CR	Yes	Yes
016	60/F	AL	MUD	BM	MAC	No									Yes	No
018	58/M	MDS/MPN	MRD	PB	MAC	No									Yes	No
019	39/M	AL	MUD	PB	MAC	Yes									Yes	No
020	48/F	MDS/MPN	MRD	PB	MAC	No	III	day 16	day 32	1	0	2	mPSL+MSC	mPSL: NC, MSC: CR	No	
021	63/M	AL	Haplo	PB	RIC	No	III	day 3	day 4	0	0	3	mPSL	CR	Yes	No
023	64/M	Lymphoma	Haplo	PB	RIC	No	III	day 4	day 5	0	0	2	mPSL	CR	No	
024	48/F	Lymphoma	CB	CB	MAC	Yes									No	
025	57/F	AL	MRD	PB	MAC	No									Yes	No
026	68/M	MDS/MPN	MUD	BM	MAC	No									No	

027	54/M	AL	CB	CB	MAC	Yes	II	day 14	day 41	2	0	1	mPSL	CR	No	
029	55/F	Lymphoma	Haplo	PB	RIC	No									Yes	No
030	67/M	MDS/MPN	Haplo	PB	RIC	No									Yes	No
031	72/M	AL	CB	CB	RIC	No	I	day 44	day 45	2	0	0	topical steroid	CR	Yes	Yes
032[§|]	63/M	MDS/MPN	Haplo	PB	RIC	Yes									Yes	Yes
034	68/F	AL	CB	CB	RIC	Yes	III	day 9	day 43	3	3	3	mPSL+MSC +ATG	NC after all treatments	Yes	No
035	46/F	Lymphoma	Haplo	PB	RIC	No	II	day 19	day 23	3	0	0	mPSL	CR	Yes	Yes
036	54/M	Lymphoma	Haplo	PB	RIC	No									No	
037	66/M	MDS/MPN	Haplo	PB	RIC	No									No	
038	58/F	AL	MUD	BM	MAC	Yes									Yes	No
039	58/F	AL	MUD	BM	MAC	No									Yes	Yes
040	55/F	AL	CB	CB	MAC	Yes									Yes	No
041	55/M	AL	MUD	PB	MAC	No	II	day 23	day 24	3	0	0	mPSL	CR	Yes	No
042	52/M	MDS/MPN	Mismatched UD	BM	MAC	No	III	day 11	day 11	2	0	3	mPSL	CR	No	
043	51/F	AL	Haplo	PB	RIC	No									Yes	Yes
045	21/F	Other disease	CB	CB	RIC	Yes	II	day 5	day 17	0	0	3	mPSL	CR	Yes	Yes
046	51/F	AL	MRD	PB	MAC	No									Yes	No
047	60/F	AL	MUD	BM	MAC	No	I	day 38	day 39	2	0	0	topical steroid	CR	Yes	Yes
048	57/F	AL	Haplo	PB	RIC	No	IV	day 3	day 5	2	4	3	mPSL+MSC	NC after all treatments	No	
050	37/M	AL	CB	CB	MAC	Yes	II	day 11	day 15	3	0	1	mPSL	CR	Yes	No
051	22/M	AL	Haplo	PB	RIC	No	I	day 35	day 35	1	0	0	topical steroid	CR	No	
052	50/M	AL	Haplo	PB	RIC	No									Yes	No
058	52/M	MDS/MPN	Haplo	PB	MAC	No	III	day 11	day 11	0	0	2	mPSL	CR	No	
062	55/M	MDS/MPN	CB	CB	MAC	Yes									No	

* All 46 cases included in our analysis are shown, and numbers assigned to excluded cases are omitted.

† Methyl prednisolone was initially administered at 1–2 mg/kg/day and then tapered for acute GVHD.

‡ All cord blood was derived from an unrelated donor.

§ Patient #32 was the same patient as #11, who was re-transplanted after underlying disease relapsed.

M: male, F: female, TBI: total body irradiation, GVHD: graft-versus-host disease, MDS/MPN: myelodysplastic syndrome/myeloproliferative neoplasms, AL: acute leukaemia, BM: bone marrow, PB: peripheral blood stem cells, CB: cord blood, MAC: myeloablative conditioning, RIC: reduced-intensity conditioning, NMA: nonmyeloablative, MRD: matched related donor, MUD: matched unrelated donor, UD: unrelated donor, Haplo: haploidentical donor, , mPSL: methylprednisolone, MSC: mesenchymal stem cell, ATG: anti-thymocyte globulin, CR: complete response, NC: no change.

Supplementary Table 3. Predictive factors of *Enterococcus* domination

The following clinical variables were assessed as univariate predictors in each patient.

Predictor	Hazard ratio (95% CI)	*P* value
**Age**		
>54.5 years (versus <54.5 years)	1.33 (0.64–2.75)	0.44
**Sex**		
female (versus male)	1.65 (0.80–3.40)	0.17
**Underlying diagnosis of acute leukaemia (versus others)**	**2.48 (1.13–5.45)**	**0.024**
**Donor source (versus matched related donor)**		
matched unrelated donor	1.20 (0.41–3.48)	0.74
cord blood	0.81 (0.29–2.24)	0.68
haploidentical donor	0.70 (0.27–1.82)	0.47
**Graft type (versus bone marrow)**		
peripheral blood stem cells	0.63 (0.25–1.61)	0.33
cord blood	0.65 (0.21–1.96)	0.44
**Conditioning regimen**		
myeloablative conditioning (versus reduced intensity conditioning)	1.20 (0.58–2.50)	0.64
**Total body irradiation-containing regimen**		
yes (versus no)	0.95 (0.43–2.07)	0.89

CI: confidence interval.
