# Supplementary material for: An enterococcal phage-derived enzyme suppresses graft-versus-host disease
Source: Nature. 2024 Jul 10;632(8023):174–81. doi: 10.1038/s41586-024-07667-8 (PMC11291292; doi:10.1038/s41586-024-07667-8)
Supplement: Supplementary file 2 — Reporting Summary [file 41586_2024_7667_MOESM2_ESM.pdf]

nature portfolio

Corresponding author(s): Seiya Imoto and Satoshi Uematsu

Last updated by author(s): May 21, 2024

Reporting Summary

Nature Portfolio wishes to improve the reproducibility of the work that we publish. This form provides structure for consistency and transparency in reporting. For further information on Nature Portfolio policies, see our Editorial Policies and the Editorial Policy Checklist.

Please do not complete any field with "not applicable" or n/a. Refer to the help text for what text to use if an item is not relevant to your study.
For final submission: please carefully check your responses for accuracy; you will not be able to make changes later.

Statistics

For all statistical analyses, confirm that the following items are present in the figure legend, table legend, main text, or Methods section.

n/a	Confirmed	
☐	☒	The exact sample size (*n*) for each experimental group/condition, given as a discrete number and unit of measurement
☐	☒	A statement on whether measurements were taken from distinct samples or whether the same sample was measured repeatedly
☐	☒	The statistical test(s) used AND whether they are one- or two-sided<br>*Only common tests should be described solely by name; describe more complex techniques in the Methods section.*
☐	☒	A description of all covariates tested
☐	☒	A description of any assumptions or corrections, such as tests of normality and adjustment for multiple comparisons
☐	☒	A full description of the statistical parameters including central tendency (e.g. means) or other basic estimates (e.g. regression coefficient) AND variation (e.g. standard deviation) or associated estimates of uncertainty (e.g. confidence intervals)
☐	☒	For null hypothesis testing, the test statistic (e.g. *F*, *t*, *r*) with confidence intervals, effect sizes, degrees of freedom and *P* value noted<br>*Give P values as exact values whenever suitable.*
☒	☐	For Bayesian analysis, information on the choice of priors and Markov chain Monte Carlo settings
☒	☐	For hierarchical and complex designs, identification of the appropriate level for tests and full reporting of outcomes
☒	☐	Estimates of effect sizes (e.g. Cohen's *d*, Pearson's *r*), indicating how they were calculated

Our web collection on statistics for biologists contains articles on many of the points above.

Software and code

Policy information about availability of computer code

Data collection

No software is used.

Data analysis

QIIME2 (version, 2018.11): https://qiime2.org
EZR (version 1.41):https://cran.r-project.org/web/packages/RcmdrPlugin.EZR/index.html
cutadapt (v.1.18): http://cutadapt.readthedocs.io/en/stable/index.html
PRINSEQ (lite v.0.20.4): http://prinseq.sourceforge.net/
SPAdes (v3.13.0): http://cab.spbu.ru/software/spades/
BayesHammer (as bundled with SPAdes v3.13.0): http://bioinf.spbau.ru/spades/bayeshammer
MetaSPAdes (v3.13.0): http://cab.spbu.ru/software/spades/
CD-HIT-EST (v.4.8): http://weizhongli-lab.org/cd-hit/
BLAST+ (v.2.5): https://blast.ncbi.nlm.nih.gov/Blast.cgi?PAGE_TYPE=BlastDocs&DOC_TYPE=Download
VirSorter (v1.0.3): https://github.com/simroux/VirSorter
MetaProdigal (v2.6.3): https://github.com/hyattpd/Prodigal
blast2lca (v.0.800): https://github.com/emepyc/Blast2lca
GHOST-MP (v.1.3.4): http://www.bi.cs.titech.ac.jp/ghostmp/index.html
PhyloPythiaS+ (v1.4): https://github.com/algbioi/ppsplus
MetaPhlAn 2.0 (v.2.5.0): http://huttenhower.sph.harvard.edu/metaphlan2
BBMap (v.38.76): https://jgi.doe.gov/data-andtools/bbtools/
BBtools (v.37.68): https://jgi.doe.gov/data-andtools/bbtools/
NCBI RefSeq nucleotide sequence (December 13, 2021): https://www.ncbi.nlm.nih.gov/nuccore/
NCBI protein (January 25, 2022): https://www.ncbi.nlm.nih.gov/protein/

KEGG (September 16, 2018): http://www.genome.jp/kegg/
MUSCLE (v3.8.31): https://www.drive5.com/muscle/
Jalview (v2.11.3.2): https://www.jalview.org
PHROG (version 4): https://phrogs.lmge.uca.fr/
ggplot2 (v.3.3.6): https://ggplot2.tidyverse.org

For manuscripts utilizing custom algorithms or software that are central to the research but not yet described in published literature, software must be made available to editors and reviewers. We strongly encourage code deposition in a community repository (e.g. GitHub). See the Nature Portfolio guidelines for submitting code & software for further information.

Data

Policy information about availability of data

All manuscripts must include a data availability statement. This statement should provide the following information, where applicable:

- Accession codes, unique identifiers, or web links for publicly available datasets
- A description of any restrictions on data availability
- For clinical datasets or third party data, please ensure that the statement adheres to our policy

All data supporting the findings of this study are provided within the manuscript and its Supplementary Information. Sequencing data generated for this study have been deposited in NCBI SRA (PRJNA1095194 and PRJNA1109929). The RefSeq protein database downloaded from the NCBI ftp site on May 5, 2020 and January 17, 2021, and NCBI RefSeq nucleotide sequence data downloaded on October 24, 2021 have been deposited in Zenodo (https://doi.org/10.5281/zenodo.11196056 and https://doi.org/10.5281/zenodo.11239382). Source data are provided with this paper.

Human research participants

Policy information about studies involving human research participants and Sex and Gender in Research.

Reporting on sex and gender	Twenty-five of 46 (54.3%) participants were male, and our results can be applied to both sexes.
Population characteristics	Patients with haematological diseases undergoing allogenic haematopoietic cell transplantation from January 2019 to June 2020 were included. The median age of patients was 54.5 years (range: 19–72), and all of them were Japanese. The patients had the following underlying diseases: 26 patients (56.5%) had acute leukaemia, 12 (26.1%) had myelodysplastic syndrome/ myeloproliferative neoplasms, 7 (15.2%) had malignant lymphoma, and 1 (2.2%) had another disease.
Recruitment	Participants were recruited before allo-HCT, and the medical team provided both verbal and written information about the research study before obtaining the written consent. Signed informed consent was obtained from each participant. No potential biases are present in our study.
Ethics oversight	This study protocol was approved by the Ethics Committee of Osaka Metropolitan University and the Institute of Medical Science, The University of Tokyo (4188 and 30-92-B0320).

Note that full information on the approval of the study protocol must also be provided in the manuscript.

Field-specific reporting

Please select the one below that is the best fit for your research. If you are not sure, read the appropriate sections before making your selection.

☒ Life sciences ☐ Behavioural & social sciences ☐ Ecological, evolutionary & environmental sciences

For a reference copy of the document with all sections, see nature.com/documents/nr-reporting-summary-flat.pdf

Life sciences study design

All studies must disclose on these points even when the disclosure is negative.

Sample size	No statistical methods were used to predetermine sample size. 46 individuals were analyzed in this study.
Data exclusions	In human data analysis, 18 of 64 patients were excluded from sample collections and data analysis due to early withdrawal from the study or rapid worsening of their condition. In animal experiments,no data was excluded.
Replication	All findings shown in Fig. 2c-e, Extended Data Fig. 2a, Extended Data Fig. 2b, Extended Data Fig. 3, and Extended Data Fig. 4 have been reproduced in two independent experiments. Other experiments were performed in indicated sample sizes.
Randomization	Randomization was not applied in human metagenome analyses and clinical evaluations. Enterococcus domination was defined as >25% of the genus Enterococcus. The first occurrence of Enterococcus domination in each case was defined as the endpoint of interest. In animal experiments, samples were randomly allocated into experimental groups.
Blinding	The number of CFUs (Fig. 3a and Extended Data Fig. 5) and survival rates (Fig. 3b, Fig. 4d, Extended Data Fig. 7, and Extended Data Fig. 8) were determined by technicians who were blinded to expected outcomes. Blinding was not relevant for metagenomic analysis, since the study was

based on objective analysis methods. Full blinding was not performed for other experiments because the treatment groups needed to be clear.

Reporting for specific materials, systems and methods

We require information from authors about some types of materials, experimental systems and methods used in many studies. Here, indicate whether each material, system or method listed is relevant to your study. If you are not sure if a list item applies to your research, read the appropriate section before selecting a response.

Materials & experimental systems

n/a	Involved in the study
☐	☒ Antibodies
☒	☐ Eukaryotic cell lines
☒	☐ Palaeontology and archaeology
☐	☒ Animals and other organisms
☒	☐ Clinical data
☒	☐ Dual use research of concern

Methods

n/a	Involved in the study
☒	☐ ChIP-seq
☒	☐ Flow cytometry
☒	☐ MRI-based neuroimaging

Antibodies

Antibodies used	His-Tag monoclonal antibody (Proteintech) (clone:1B7G5, Cat no:66005-1-Ig, 1:2000)<br>Goat Anti-Mouse IgG (H+L) (Jachson ImmunoResearch) (Cat no:115-035-003, 1:10000)
Validation	All the antibodies are commercially available. Quality validations were performed by each manufacturer. Validation statements are provided on the manufacturer's website.<br>His-Tag monoclonal antibody (Proteintech): https://www.ptglab.co.jp/products/His-Tag-Antibody-66005-1-Ig.htm<br>Goat Anti-Mouse IgG (H+L) (Jachson ImmunoResearch): https://www.jacksonimmuno.com/catalog/products/115-035-003

Animals and other research organisms

Policy information about studies involving animals; ARRIVE guidelines recommended for reporting animal research, and Sex and Gender in Research

Laboratory animals	Germ-free C57BL/6 (H2kb) and specific-pathogen-free 129SvJ/JmsSlc mice (6–8 weeks old) were purchased from SLC Japan and CLEA Japan respectively and were used for the indicated experiments.
Wild animals	This study did not involve wild animals.
Reporting on sex	In acute graft-versus-host disease mouse models, our results can be only applied to female sex because we used female donor and recipient mice.
Field-collected samples	This study did not involve samples collected from the field.
Ethics oversight	All animal experiments were performed with the approval of the Animal Care and Use Committees of Osaka Metropolitan University.

Note that full information on the approval of the study protocol must also be provided in the manuscript.

This checklist template is licensed under a Creative Commons Attribution 4.0 International License, which permits use, sharing, adaptation, distribution and reproduction in any medium or format, as long as you give appropriate credit to the original author(s) and the source, provide a link to the Creative Commons license, and indicate if changes were made. The images or other third party material in this article are included in the article's Creative Commons license, unless indicated otherwise in a credit line to the material. If material is not included in the article's Creative Commons license and your intended use is not permitted by statutory regulation or exceeds the permitted use, you will need to obtain permission directly from the copyright holder. To view a copy of this license, visit http://creativecommons.org/licenses/by/4.0/
